# Identification and validation of COL6A1 as a novel target for tumor electric field therapy in glioblastoma

**DOI:** 10.1111/cns.14802

**Published:** 2024-06-17

**Authors:** Junyi Chen, Yuyang Liu, Jinxin Lan, Hongyu Liu, Qingyun Tang, Ze Li, Xiaoguang Qiu, Wentao Hu, Jiaxin Xie, Yaping Feng, Lilin Qin, Xin Zhang, Jialin Liu, Ling Chen

**Affiliations:** ^1^ Medical School of Chinese PLA Beijing China; ^2^ Department of Neurosurgery Chinese PLA General Hospital Beijing China; ^3^ Department of Neurosurgery 920th Hospital of Joint Logistics Support Force Kunming China; ^4^ School of Medicine Nankai University Tianjin China; ^5^ Department of Neurosurgery Hainan Hospital of Chinese PLA General Hospital Hainan China; ^6^ Department of Gastroenterology 920th Hospital of Joint Logistics Support Force Kunming China; ^7^ Beijing Tiantan Hospital, Capital Medical University Beijing China; ^8^ Zhejiang Cancer Hospital Zhejiang Hangzhou China

**Keywords:** COL6A1, extracellular matrix, glioblastoma multiforme, tumor electric field therapy

## Abstract

**Background:**

Glioblastoma multiforme (GBM) is the most aggressive primary brain malignancy. Novel therapeutic modalities like tumor electric field therapy (TEFT) have shown promise, but underlying mechanisms remain unclear. The extracellular matrix (ECM) is implicated in GBM progression, warranting investigation into TEFT‐ECM interplay.

**Methods:**

T98G cells were treated with TEFT (200 kHz, 2.2 V/m) for 72 h. Collagen type VI alpha 1 (COL6A1) was identified as hub gene via comprehensive bioinformatic analysis based on RNA sequencing (RNA‐seq) and public glioma datasets. TEFT intervention models were established using T98G and Ln229 cell lines. Pre‐TEFT and post‐TEFT GBM tissues were collected for further validation. Focal adhesion pathway activity was assessed by western blot. Functional partners of COL6A1 were identified and validated by co‐localization and survival analysis.

**Results:**

TEFT altered ECM‐related gene expression in T98G cells, including the hub gene COL6A1. COL6A1 was upregulated in GBM and associated with poor prognosis. Muti‐database GBM single‐cell analysis revealed high‐COL6A1 expression predominantly in malignant cell subpopulations. Differential expression and functional enrichment analyses suggested COL6A1 might be involved in ECM organization and focal adhesion. Western blot (WB), immunofluorescence (IF), and co‐immunoprecipitation (Co‐IP) experiments revealed that TEFT significantly inhibited expression of COL6A1, hindering its interaction with ITGA5, consequently suppressing the FAK/Paxillin/AKT pathway activity. These results suggested that TEFT might exert its antitumor effects by downregulating COL6A1 and thereby inhibiting the activity of the focal adhesion pathway.

**Conclusion:**

TEFT could remodel the ECM of GBM cells by downregulating COL6A1 expression and inhibiting focal adhesion pathway. COL6A1 could interact with ITGA5 and activate the focal adhesion pathway, suggesting that it might be a potential therapeutic target mediating the antitumor effects of TEFT.

## Graphical abstract

Innovative TEFT therapeutic target COL6A1 in GBM.

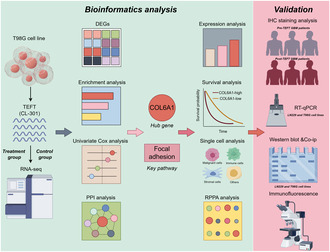

## 1 INTRODUCTION

Glioblastoma multiforme (GBM) represents the most aggressive malignant primary brain tumor, accounting for 50.1% of all intracranial neoplasms, with a 5‐year survival rate of only 6.9%.^1^ The current standard of care for GBM consists of surgical resection followed by concomitant radiotherapy and alkylating chemotherapy with temozolomide (TMZ), succeeded by sixcycles of adjuvant TMZ.^2^, ^3^ However, over the past two decades, therapeutic advancements for GBM have been modest, with median overall survival (OS) of around 14.6 months and progression‐free survival of 7–10 months; nearly 100% of patients experience tumor recurrence.^2^, ^4^ Therefore, to overcome current limitations in GBM treatment, there is an urgent need to explore more efficacious and well‐tolerated novel therapeutic modalities for this disease.

Tumor electric field therapy (TEFT) represents the only innovative modality approved and incorporated into the National Comprehensive Cancer Network (NCCN) guidelines for GBM in the past decade.^5^, ^6^ It utilizes low‐intensity (1–3 V/cm), intermediate‐frequency (100–300 kHz), alternating electric fields to generate non‐uniform fields that disrupt late‐stage mitotic spindle formation during cytokinesis, causing cell cycle arrest and apoptosis of tumor cells.^7^, ^8^, ^9^ As such, TEFT exerts potent inhibitory effects on highly proliferative cells, with minimal impact on normal tissues.^10^ The antiproliferative effects of alternating electric fields were first discovered by Kirson et al. in 2004, and subsequent transplantation of treated tumor cells into mice impeded tumor growth.^11^ Subsequent in vitro and in vivo studies, together with clinical investigations, have validated the safety and efficacy of TEFT for restraining tumor progression. Our previous research found that in vitro, TEFT inhibited tumor cell viability, proliferation, and invasion in a frequency‐ and intensity‐dependent manner, with random‐sequence fields exhibiting superior antitumor effects over unidirectional fields.^12^ Our previous research also found that in mouse models, TEFT slowed tumor growth and prolonged survival without significant adverse reactions except for local contact dermatitis.^12^, ^13^ A phase III trial in recurrent GBM demonstrated comparable efficacy of TEFT monotherapy to chemotherapy with better quality of life.^14^ Another phase III trial revealed the combination of TEFT and TMZ maintenance therapy after chemoradiation conferred progression‐free and OS benefits in newly diagnosed GBM, with progression‐free survival (PFS) prolonged to 6.7 months and median OS to 20.9 months, which are clinically meaningful improvements in GBM treatment.^15^ Based on this evidence, TEFT was granted Food and Drug Administration (FDA) approval in 2011 and 2015 for treating recurrent and newly diagnosed GBM, respectively, and later in 2019 for unresectable malignant pleural mesothelioma.^16^ With the increasing adoption of TEFT, growing research efforts have focused on elucidating its antitumor mechanisms, which remain incompletely defined and warrant further investigation.

The extracellular matrix (ECM) comprises non‐cellular components present throughout all body organs and tissues, primarily composed of interstitial fluid, proteins, and polysaccharides.^17^ In addition to providing physical scaffolding and protection, the ECM participates in various biological processes including cell proliferation, differentiation, invasion, and migration.^18^ Moreover, the ECM is a dynamic structure that undergoes constant tissue renewal and remodeling in response to relevant stimuli.^18^ Importantly, the tumor ECM plays a pivotal role, where alterations in its biophysical properties and signaling pathways can promote cancer cell survival, proliferation, and invasive phenotypes like chemoresistance.^19^ The ECM can also facilitate tumor metastasis, as dormant tumor cells upon reactivation are capable of remodeling the ECM to support colonization.^20^, ^21^ Furthermore, the ECM represents a potential therapeutic target and is implicated in cancer diagnosis and prognosis.^22^, ^23^ In GBM, complex interactions among tumor cells, normal brain cells (neurons and astrocytes), and the ECM contribute to persistent tumor infiltration and treatment failure.^24^ Therefore, comprehensively elucidating the functional roles of the ECM in GBM is imperative.

Recent studies have shown that tumor‐treating electric fields (TEFT) may exert antitumor effects by influencing the ECM components of the tumor microenvironment (TME).^25^, ^26^ As an important part of the TME, TEFT treatment may regulate the expression patterns of ECM proteins synthesized by tumor cells, inhibit ECM‐mediated pro‐carcinogenic signaling transduction, and ultimately suppress tumor invasion and metastasis.^27^, ^28^, ^29^ Our study found that after screening a range of ECM‐related proteins, collagen type VI alpha 1 (COL6A1) emerged as a key upregulated gene in GBM tissues. Its high expression was associated with poor prognosis in patients. We then examined how TEFT treatment affected ECM‐related gene expression in GBM cells. We found that TEFT significantly downregulated COL6A1 expression in GBM tissues. This suggested that TEFT could promote tumor ECM remodeling, and COL6A1 might play a central role in mediating this effect. By modulating COL6A1, TEFT may inhibit critical ECM signals that drive GBM progression. Overall, our findings indicate that COL6A1 may be a promising new therapeutic target for TEFT treatment in GBM patients.

## 2 METHODS

### 2.1 Cultures of GBM cell line

The T98G and Ln229 cell lines used were purchased from the Institute of Basic Medicine at China Medical College. Dulbecco's modified Eagle's medium high‐glucose (DMEM, Gibco)‐containing 10% fetal bovine serum (FBS, Gibco) was used to culture the cells. Cell culture was constructed in an incubator with conditions set at 37°C and 5% CO_2_.

### 2.2 Tumor electric field treatment on cells

Tumor cells were cultured on 20‐mm‐diameter glass slides (Nest 801008) in specialized cell culture vessels obtained from Antai Kangcheng Biotechnology Co., Ltd. Cells were suspended at a density of 2 × 10^5^ cells/mL and 150 μL of the suspension was seeded onto each slide. Seeded cells were incubated overnight at 37°C with 5% CO_2_ to allow adhesion. Electric field treatment was administered using a TEFT device developed by our research group (TEFT, CL‐301A). Treatment groups underwent TEFT exposure with field parameters set to 200 kHz frequency and 2.2 V/m field strength in a fixed sequence mode for 72 hours, with the relevant parameters having been validated in previous experiments.^12^ Control groups were maintained under identical culture conditions without TEFT exposure.

### 2.3 Transcriptional sequencing of T98G cells

Cells were processed for total RNA isolation following the manufacturer's protocol using Trizol reagent from ThermoFisher. Polyadenylated (poly(A)) RNAs and non‐coding RNAs (ncRNAs) were purified from total RNA using oligo(dT) beads. RNA sequencing (RNA‐seq) libraries were constructed using the enriched RNAs and sequenced on an Illumina platform at Majorbio Corporation. Raw sequencing reads were preprocessed by trimming adapter sequences and filtering out low‐complexity and low‐quality reads. Clean reads were then aligned to the GRCh38.p13 human reference genome assembly using HISAT2 aligner. The raw and processed RNA sequencing data from this study were submitted to the NCBI Gene Expression Omnibus (GEO) database.

### 2.4 Acquisition of GBM public data

Gene expression and matched clinical data for glioma samples were obtained from the GlioVis portal (http://gliovis.bioinfo.cnio.es/), including 620 gliomas from The Cancer Genome Atlas (TCGA) and 315 gliomas from the Rembrandt glioma cohort. Normalization of raw data was constructed by the GlioVis portal automatically. Normal brain tissue expression data were downloaded from the genotype tissue expression (GTEx) project via the UCSC Xena browser (https://xenabrowser.net/datapages/). Immunohistochemical (IHC) staining of glioma tissue sections of GBM cell lines was accessed from the Human Protein Atlas (HPA) database (https://www.proteinatlas.org). Reverse‐phase protein array (RPPA) data for TCGA‐GBM specimens were also retrieved from GlioVis.

### 2.5 Analysis of T98G transcriptional sequencing data

Principal component analysis (PCA) was performed using the ggplot2 R package (version 4.2.1) to assess sample similarity based on gene expression data. Heatmaps were generated with ggplot2 (version 3.3.6) to visualize expression profiles. Identification of differentially expressed genes (DEGs) was carried out using DESeq2 (version 1.36.0) package. DEGs were defined as absolute log_2_ fold change >2 and adjusted *p* < 0.05. Genes with log_2_ fold change >2 were defined as upregulated DEGs (indicating increased expression). Genes with log_2_ fold change <2 were defined as downregulated DEGs (indicating decreased expression). The R package (version 4.4.4) was used to perform enrichment analysis of the DEGs for Gene Ontology (GO) terms and Kyoto Encyclopedia of Genes and Genomes (KEGG). The pathway map of focal adhesion was downloaded from KEGG database (https://www.kegg.jp/).

### 2.6 Analysis of the hub gene related to TEFT‐induced biological process

DEGs belonging to the extracellular matrix organization Gene Ontology term as well as prognosis‐related genes in TCGA GBM were identified. Among these, DEGs associated with prognosis were further filtered using the Venn diagram, retaining downregulated DEGs with hazard ratio (HR) >1 and upregulated DEGs with HR < 1. The protein–protein interaction (PPI) network was constructed and CytoHubba analysis was performed to identify hub genes related to the extracellular matrix organization process induced by TEFT.

### 2.7 COL6A1‐related single‐cell analysis in GBM dataset

The Tumor Immune Single‐cell Hub 2 (TISCH2) database (http://tisch.comp‐genomics.org/) was accessed to obtain single‐cell RNA sequencing data for GBM. Analysis of these data was performed to examine the expression pattern of COL6A1 gene across the various GBM cell subpopulations. Dimensionality reduction with uniform manifold approximation and projection (UMAP) was performed on the aggregated single‐cell expression data. Violin plots were then generated to visualize and compare COL6A1 expression levels between the identified GBM cell clusters.

### 2.8 Identification of potential functions of COL6A1 in GBM

To investigate the potential functional roles of COL6A1 in GBM, DEGs and pathway enrichment analyses were performed using TCGA‐GBM and Rembrandt‐GBM datasets. Patients were stratified into high‐ (high‐expression group: 50%–100%) and low (low‐expression group: 0%–50%)‐COL6A1 expression groups. DEGs between groups were identified, applying filters of absolute log_2_ fold change >1 and adjusted *p* < 0.05. UpSet plot visualization was used to find DEGs in common between the two cohorts. GO and KEGG pathway enrichment of DEGs was conducted using the clusterProfiler R package. At the protein level, differential expression analysis between COL6A1‐high and ‐low groups was carried out using RPPA data from TCGA GBM based on the GlioVis portal. To improve result robustness, the intersection of differential proteins identified across three independent RPPA datasets (HG‐U133A, Agilent‐4502A, and RNA‐seq) was chosen.

### 2.9 GBM sample collection

This study received ethical approval from the Institutional Review Board of PLA General Hospital, with batch number S2018‐089‐01. All participating patients provided their informed consent. Three paired paraffin‐embedded GBM tissues obtained from patients before and after TEFT were used for IHC staining.

### 2.10 IHC staining

The tissues were fixed in 4% paraformaldehyde solution, embedded in paraffin, and sectioned into 4‐μm‐thick slices. The tissue sections were mounted on slides and processed following the previously described protocol.^12^ The tissue sections were incubated overnight at 4°C with a primary antibody against COL6A1 (Abcam, ab151422, 1:1000 dilution) in 1% goat serum (Balb, WE0320) PBS solution. After washing, the sections were incubated for 1 h at room temperature with the appropriate secondary antibody. The tissue slices were then stained using the ABC Horseradish Peroxidase kit (Vector Laboratories) and 3,3'‐diaminobenzidine (DAB) as the chromogen for visualization. Hematoxylin counterstaining was performed to visualize nuclei. Two pathologists, blinded to clinical information on the samples, independently evaluated and scored the resulting immunohistochemical staining patterns.

### 2.11 Construction of stable COL6A1 knockdown cell lines

To construct stable knockdown of the COL6A1 gene in the GBM cell lines (T98G and Ln229), a lentivirus‐mediated shRNA knockdown method was employed. The shRNA sequences targeting the COL6A1 gene were specifically designed and synthesized by Tsingke Biotechnology Company (Beijing). The shRNA was cloned into a lentiviral vector plasmid, and HEK293T cells were transfected with the plasmid using Lipofectamine 2000 (Invitrogen, 11668‐019) to package the viral particles. The virus‐containing supernatant was collected and used to infect T98G and Ln229 cells. According to the manufacturer's instructions, the culture medium was replaced with medium containing 2 μg/mL puromycin 24 h after infection for selection. The cells were cultured continuously for 2–3 weeks until single‐cell colonies appeared. After expansion, total RNA and protein were extracted, and the knockdown efficiency of the COL6A1 gene was detected by real‐time quantitative PCR (RT‐qPCR) and Western blot (WB). Single‐cell clones with significantly reduced COL6A1 gene expression were selected, yielding stable COL6A1 knockdown GBM cell lines. The shRNA sequences used can be found in Table S1.

### 2.12 COL6A1 recombinant protein addition assay

To simulate the effect of overexpression of extracellular matrix protein COL6A1 on GBM cell lines, referring to previous experiments^30^ and vendor instructions, the cells in good growth condition were divided into an experimental group and a control group. In the experimental group, exogenous human recombinant protein COL6A1 (rCOL6A1) (6 μg/mL) (Proteintech, Ag10288) was added to the cell culture medium, while in the control group, the same volume of culture medium without rCOL6A1 was added. After 48 h of culture, samples were collected for WB analysis.

### 2.13 Quantitative RT‐PCR

Total RNA was extracted from cell samples using the RNeasy Mini Kit (Qiagen). RNA was reverse transcribed into cDNA using SuperScript III Reverse Transcriptase (Invitrogen) and random hexamer primers. Real‐time quantitative PCR was performed on the Applied Biosystems 7500 Real‐Time PCR System. The PCR cycling conditions were as follows: 95°C for 10 min; followed by 40 cycles of 95°C for 15 s and 60°C for 1 min. Each reaction was run in triplicate, using β‐actin as the reference gene. Relative expression levels of the target genes compared to the reference gene were calculated using the 2‐ΔΔCt method. Primer sequences used can be found in Table S2.

### 2.14 Western blot analysis

Total protein was extracted from samples using RIPA lysis buffer (Beyotime, China) supplemented with protease inhibitor cocktail (MCE, China). Extracted proteins were separated by SDS‐PAGE using 4%–12% polyacrylamide gels and transferred to polyvinylidene difluoride (PVDF) membranes. The membranes were incubated in blocking buffer (5% skim milk in Tris‐buffered saline with 0.1% Tween 20) for 1 h at room temperature followed by overnight incubation at 4°C with primary antibodies at 1:800 dilution targeting proteins of interest. After washing with TBST, membranes were incubated for 1 h at room temperature with horseradish peroxidase‐conjugated secondary antibodies specific to the primary antibodies at 1:5000 dilution. Protein bands were visualized using enhanced chemiluminescence reagent and their densities were quantified by densitometric analysis in ImageJ software (version 1.54d). Antibodies used can be found in Table S3.

### 2.15 Prediction and validation of COL6A1 functional partners

The map of focal adhesion pathway was retrieved from KEGG database^31^ (https://www.kegg.jp/). Functional partners of COL6A1 were explored using STRING database with the default parameters and predictive scores were directly obtained from the website. Spearman correlation analysis was utilized to uncover relevance between COL6A1 and candidate functional partners. Survival analysis was used to detect the prognostic value of COL6A1 functional partner.

### 2.16 Immunofluorescence staining

T98G cells were harvested during log phase growth. After trypsinization to detach adhered cells, the cell suspension was centrifuged and the cell pellet was resuspended in fresh medium. The cells were counted and seeded onto laser confocal culture dishes (Nest, cat. No. 801001) at the appropriate density. The cells were incubated for 3 h to allow adhesion. Then, cultured overnight in 1 mL of medium added after the adhesion period. The culture medium was aspirated and the cells were washed with PBS. The cells were then fixed at room temperature for 15 min using 4% paraformaldehyde solution. The cells were permeabilized with 0.3% Triton X‐100 solution for 20 min followed by blocking with 5% BSA for 1 h. Primary antibody incubation was performed overnight at 4°C using a 1:200 dilution. After washing, the cells were incubated for 1.5 h at room temperature with fluorophore‐conjugated secondary antibody diluted at 1:500, while protected from light. Nuclei staining was done using DAPI for 10 min in the dark. Finally, the prepared cell samples were imaged under a laser‐scanning confocal microscope (Olympus, FV1000).

### 2.17 Co‐immunoprecipitation (Co‐IP)

Cell lysates were prepared in ice‐cold RIPA buffer and cleared by centrifugation. Five‐hundred microgram of lysate was incubated with 2 μg of the indicated antibody and 20 μL of protein G agarose beads (Invitrogen) overnight at 4°C with rotation. Beads were washed five times with RIPA buffer. Bound proteins were eluted by boiling in SDS sample buffer. For IgG controls, normal rabbit IgG (Santa) was substituted for the primary antibody. WB was used to study the immunoprecipitated proteins.

### 2.18 Statistical analysis

The Shapiro–Wilk test was utilized to assess the normality of the data distribution. For continuous variables following a normal distribution, the Student's *t*‐test was utilized for comparisons between two groups. For non‐normally distributed continuous data, the non‐parametric Wilcoxon rank‐sum test was used for group comparisons. The Kruskal–Wallis test followed by post‐hoc Dunn's multiple comparisons was utilized for comparisons across multiple groups. Comparison of Kaplan–Meier survival curves was accomplished using the log‐rank test. Patients were stratified into high‐ (high‐expression group: 50%–100%) and low (low‐expression group: 0%–50%)‐expression groups based on median mRNA levels. The survival R package (version 3.3.1) was utilized for survival statistics and survminer (version 3.3.6) for visualization. R software (version 4.2.1) was utilized to perform the statistical analyses. *p*‐values less than 0.05 from two‐sided tests were considered statistically significant.

## 3 RESULTS

### 3.1 Exploration of potential mechanisms underlying the antitumor effects of TEFT

**FIGURE 1:**
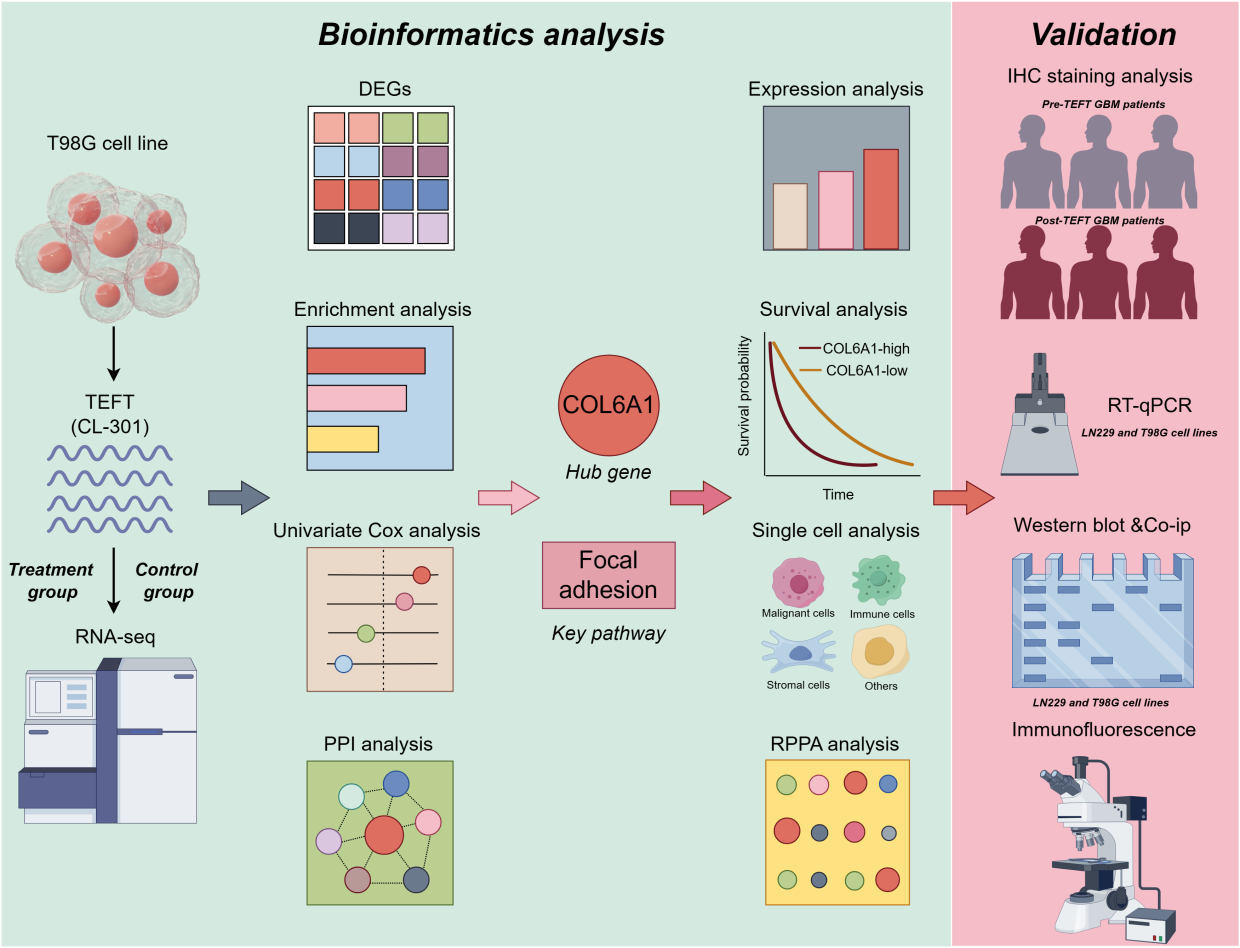
Flow chart of the study (by Figdraw).

**FIGURE 2:**
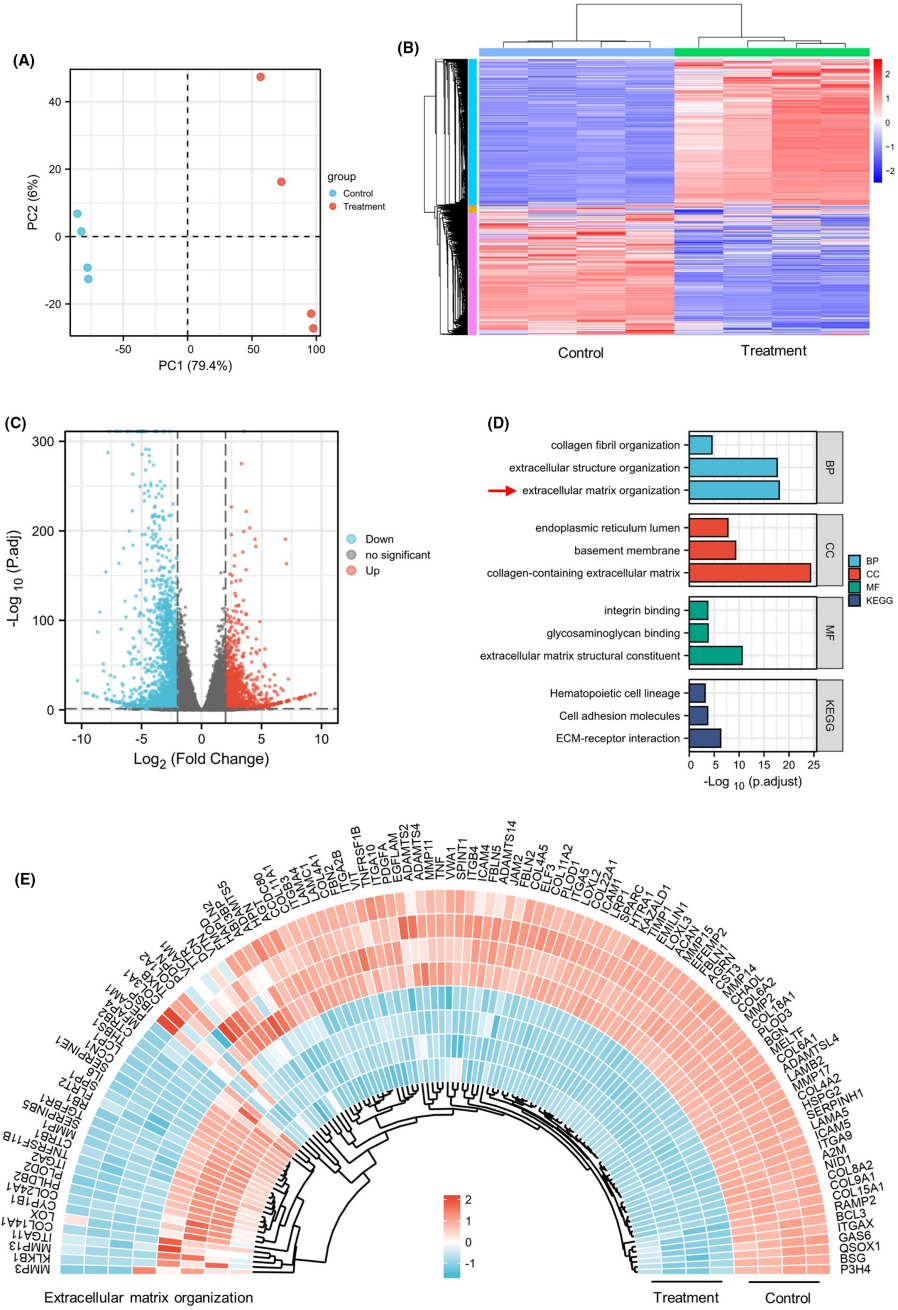
Transcriptomic profiling revealed differences between control and TEFT treatment groups. (A) PCA map showed different distribution characteristics of control and treatment groups. (B) Hierarchical clustering analysis exhibited mRNA expression features of control and treatment groups. (C) Volcano plot depicted DEGs between the two groups. (D) Enrichment analysis of DEGs. (E) Heatmap shows relative expression levels in extracellular matrix organization‐related genes.

Flow chart of the study is shown in Figure 1. PCA revealed clear separation between the control and TEFT treatment groups into two distinct clusters, indicating substantial transcriptional differences (Figure 2A). Similarly, hierarchical clustering analysis segregated the two groups based on their transcriptomic profiles (Figure 2B). Volcano plot visualization identified numerous DEGs between the groups, including 7280 upregulated and 6824 downregulated genes (Figure 2C). Enrichment analysis demonstrated that the DEGs were significantly enriched in Gene Ontology terms related to extracellular matrix organization, collagen‐containing extracellular matrix, integrin binding, extracellular matrix structural constituents, cell adhesion molecules, and ECM–receptor interactions, suggesting a close link between TEFT's antitumor effects and extracellular matrix remodeling in GBM (Figure 2D). Among these terms, extracellular matrix organization was most significant. Hierarchical clustering heatmaps of the DEGs belonging to this category revealed marked alterations in many extracellular matrix‐related genes (Figure 2E), further indicating that TEFT's antitumor effects are associated with extracellular matrix remodeling in GBM.

### 3.2 COL6A1 represented a core gene associated with TEFT‐induced extracellular matrix remodeling

**FIGURE 3:**
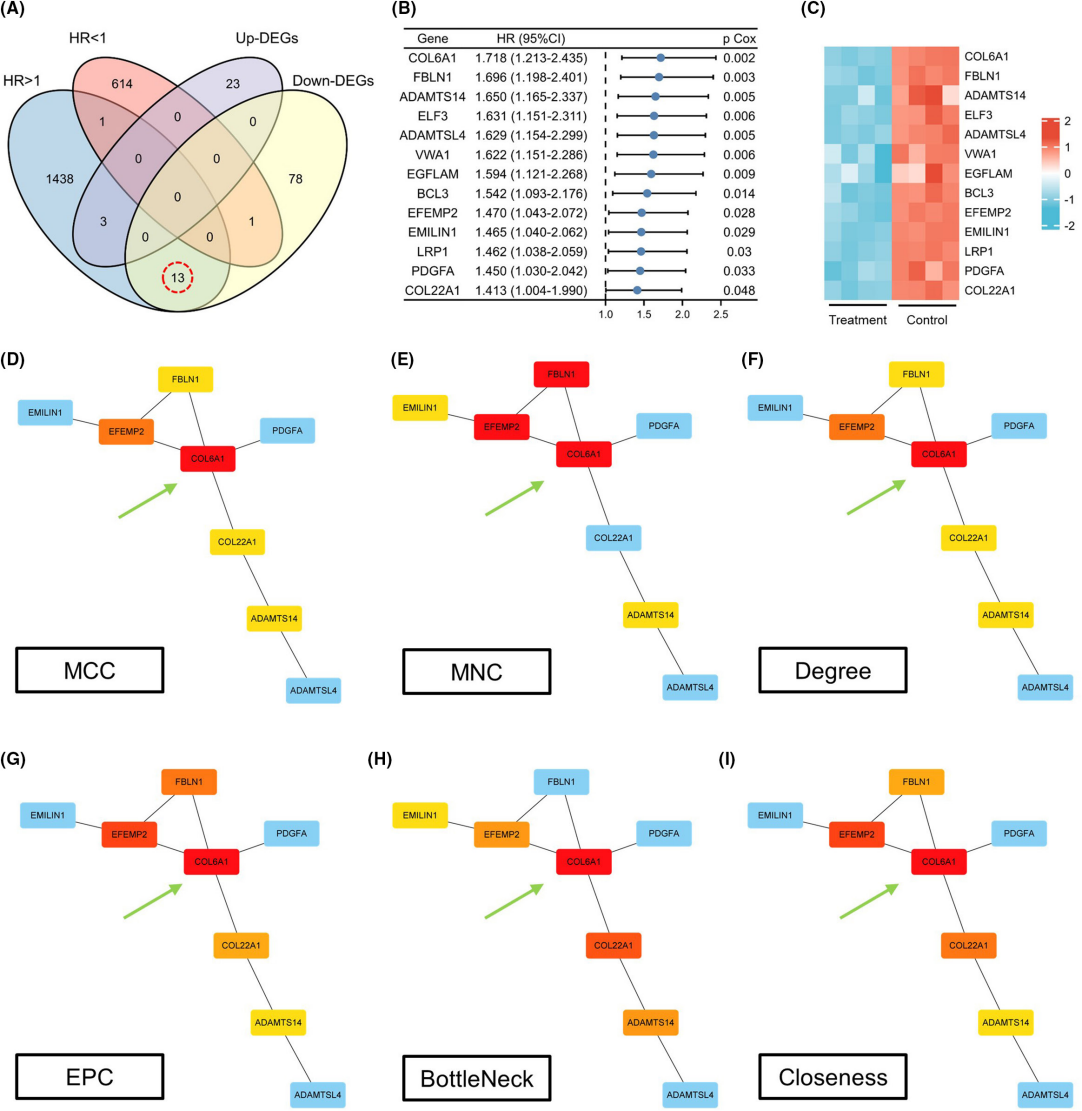
COL6A1 represented a hub gene associated with TEFT‐induced extracellular matrix remodeling. (A) The Venn diagram reveals 13 DEGs associated with extracellular matrix organization and GBM prognosis. (B) The survival forest plot showed the 13 overlapping genes correlating with poor‐prognosis, univariate Cox regression analysis. (C) The heatmap showed downregulation of the 13 overlapping genes after TEFT treatment. (D–I) PPI networks of the 13 genes and CytoHubba centrality analysis indicating COL6A1 as the top hub gene.

To identify key biological processes and associated genes underlying the antitumor effects of TEFT, we performed a series of analyses on DEGs belonging to the “extracellular matrix organization” Gene Ontology term. A Venn diagram revealed 13 prognosis‐related differentially expressed genes (Figure 3A). A survival forest plot illustrated that these 13 genes were associated with poor prognosis in GBM (Figure 3B). Heatmap analysis showed significant downregulation of these genes in the TEFT treatment group, suggesting TEFT antitumor effects are closely linked to extracellular matrix remodeling in GBM (Figure 3C). Protein–protein interaction network and CytoHubba analysis indicated COL6A1 as the top‐ranked hub gene across six algorithms, implying a crucial role for COL6A1 within this biological process (Figure 3D–I).

### 3.3 Prognostic gene COL6A1 was significantly upregulated in GBM

**FIGURE 4:**
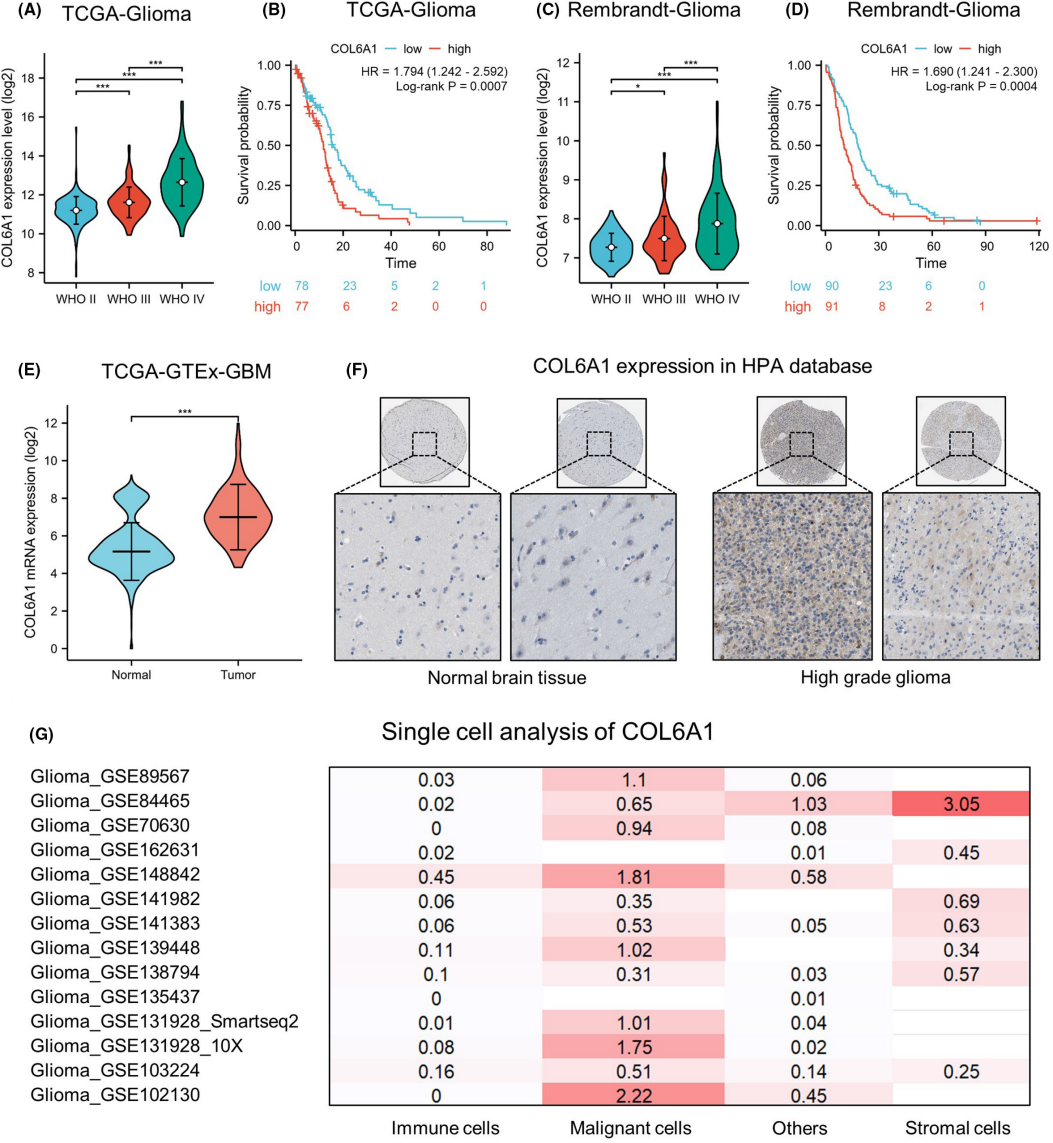
COL6A1 was significantly upregulated in GBM and indicated poor prognosis. (A) COL6A1 mRNA levels were progressively increased with higher grade in TCGA glioma dataset. Data are mean ± standard deviation (SD), ***p < 0.001, Kruskal–Wallis test, and Dunn's post‐hoc test. (B) High‐COL6A1 expression associated with shorter overall survival in TCGA glioma cohort, log‐rank test. (C) COL6A1 mRNA levels were progressively increased with higher grade in Rembrandt glioma dataset. Data are mean ± SD, *p < 0.05, ***p < 0.001, Kruskal–Wallis test, and Dunn's post‐hoc test. (D) High‐COL6A1 expression associated with shorter overall survival in Rembrandt glioma cohort, log‐rank test. (E) The COL6A1 mRNA level was markedly overexpressed in TCGA GBM compared to normal brain tissues (GTEX database). Data are mean ± SD, *p < 0.05, ***p < 0.001, Wilcox rank‐sum test. (F) IHC staining showed pronounced elevation of COL6A1 protein in HGG versus normal brain tissue in HPA database. (G) Heatmaps show upregulated COL6A1 expression on malignant cells in GBM datasets via the TISCH2 database.

Our analyses of TCGA and Rembrandt public datasets revealed that COL6A1 mRNA levels were markedly upregulated with increasing World Health Organization (WHO) grades of glioma (TCGA glioma: WHO IV vs. WHO II, ***p < 0.001, WHO IV vs. WHO III, ***p < 0.001, and WHO III vs. WHO II, ***p < 0.001; Rembrandt glioma: WHO IV vs. WHO II, ***p < 0.001, WHO IV vs. WHO III, ***p < 0.001, and WHO III vs. WHO II, *p < 0.05, Figure 4A,C). Moreover, Kaplan–Meier survival analyses demonstrated that glioma patients with high‐COL6A1 expression had significantly shorter OS (TCGA glioma: hazard ratio = 1.794, 95% confidence interval = 1.242–2.592, *p* < 0.001, Figure 4B; Rembrandt–Glioma: hazard ratio = 1.690, 95% confidence interval = 1.241–2.300, *p* < 0.001, Figure 4D). We also found COL6A1 was significantly overexpressed in GBM compared to the normal brain tissue (*p* < 0.0001, Figure 4E). Additionally, IHC results exhibited elevation of COL6A1 protein in high‐grade gliomas relative to normal brain tissue (Figure 4F). Taken together, these results implicate important roles of COL6A1 in GBM pathogenesis and its high expression associated with increased tumor malignancy and poor prognosis. COL6A1 may represent a potential biomarker and therapeutic target for GBM.

### 3.4 COL6A1 was predominantly expressed in GBM tumor cells

Multi‐database single‐cell analysis heatmaps demonstrated upregulated expression of COL6A1 in GBM tumor cells (Figure 4G). Analysis of dataset GSE148842 identified six clusters of cells (AC‐like malignant, CD8Tex, malignant, mono/macro, oligodendrocyte, and others), with violin plots and expression‐level graphs indicating high‐COL6A1 expression in AC‐like malignant and malignant cells (Figure S1A–C). Analysis of dataset GSE131928 revealed eight clusters of cells (AC‐like malignant, CD8Tex, MES‐like malignant, malignant, mono/macro, NPC‐like malignant, OPC‐like malignant, and oligodendrocyte), with violin plots and expression‐level graphs showing elevated COL6A1 levels in AC‐like malignant, MES‐like malignant, malignant, and OPC‐like malignant cells (Figure S1D–F). Together, these data indicated that COL6A1 was mainly expressed in GBM tumor cells, and TEFT might exert its antitumor effects by suppressing COL6A1 expression.

### 3.5 Investigated the potential functions of COL6A1 in GBM

**FIGURE 5:**
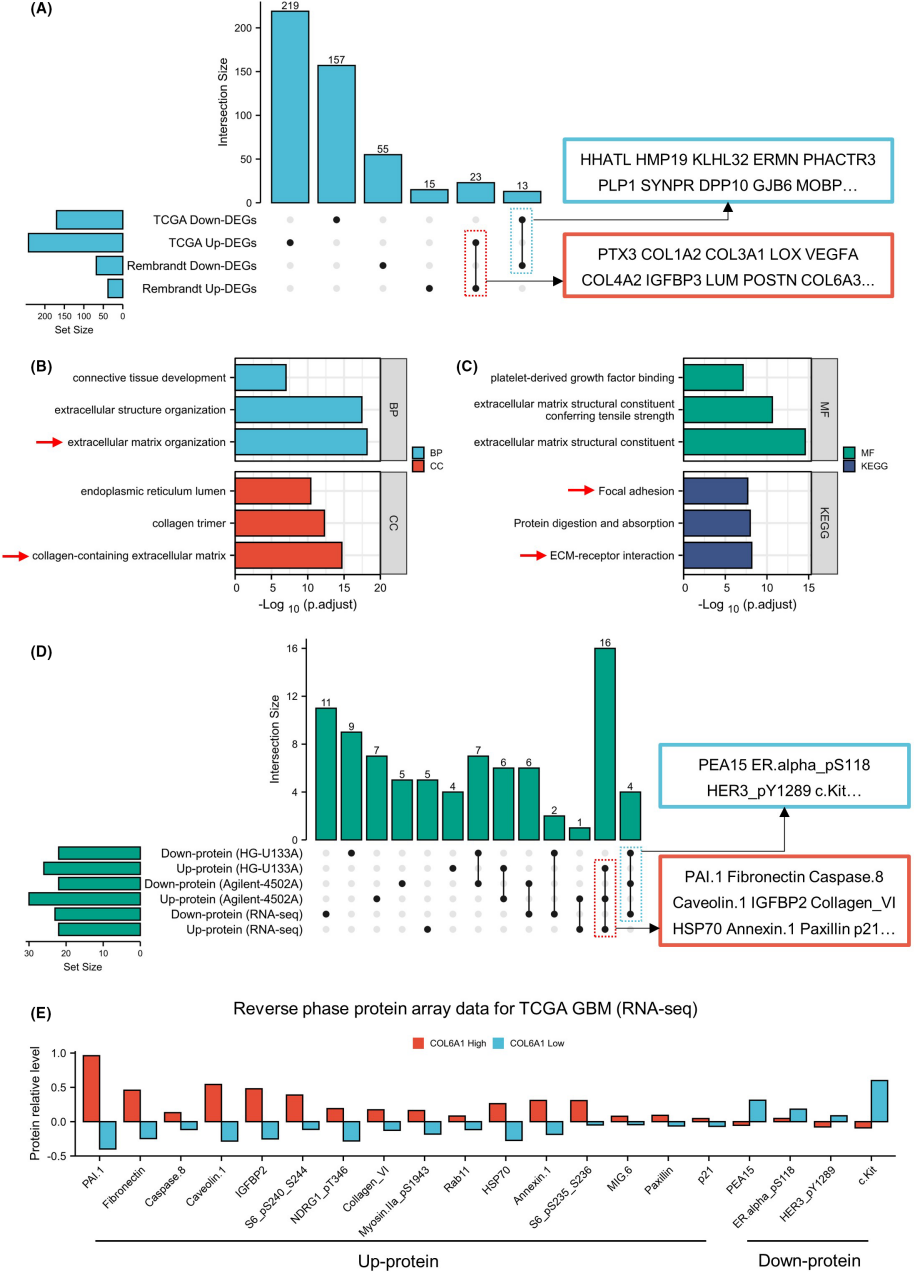
COL6A1 correlated with ECM remodeling and related protein expression in GBM. (A) Upset plot showed the overlapping DEGs from two GBM databases. (B, C) Enrichment analyses of overlapping DEGs. (D, E) RPPA analysis exhibited differentially expressed protein between COL6A1‐high and ‐low groups.

Based on the differential analysis and enrichment results between COL6A1‐high and ‐low groups in GBM cohorts, COL6A1 may be closely associated with extracellular matrix organization, collagen‐containing extracellular matrix, focal adhesion, and ECM–receptor interaction in GBM (Figure 5A–C). RPPA analysis showed that the COL6A1‐high group had higher levels of PAI.1, fibronectin, caveolin 1, and IGFBP2 proteins compared to the low‐expression group. In addition, PEA15, ER.alpha_pS118, HER3_pY1289, and c.Kit proteins were downregulated in the COL6A1‐high group (Figure 5D,E). These results suggested that COL6A1‐high group may possess unique extracellular matrix characteristics and deserved further exploration.

### 3.6 TEFT markedly suppressed COL6A1 expression and focal adhesion pathway activity

**FIGURE 6:**
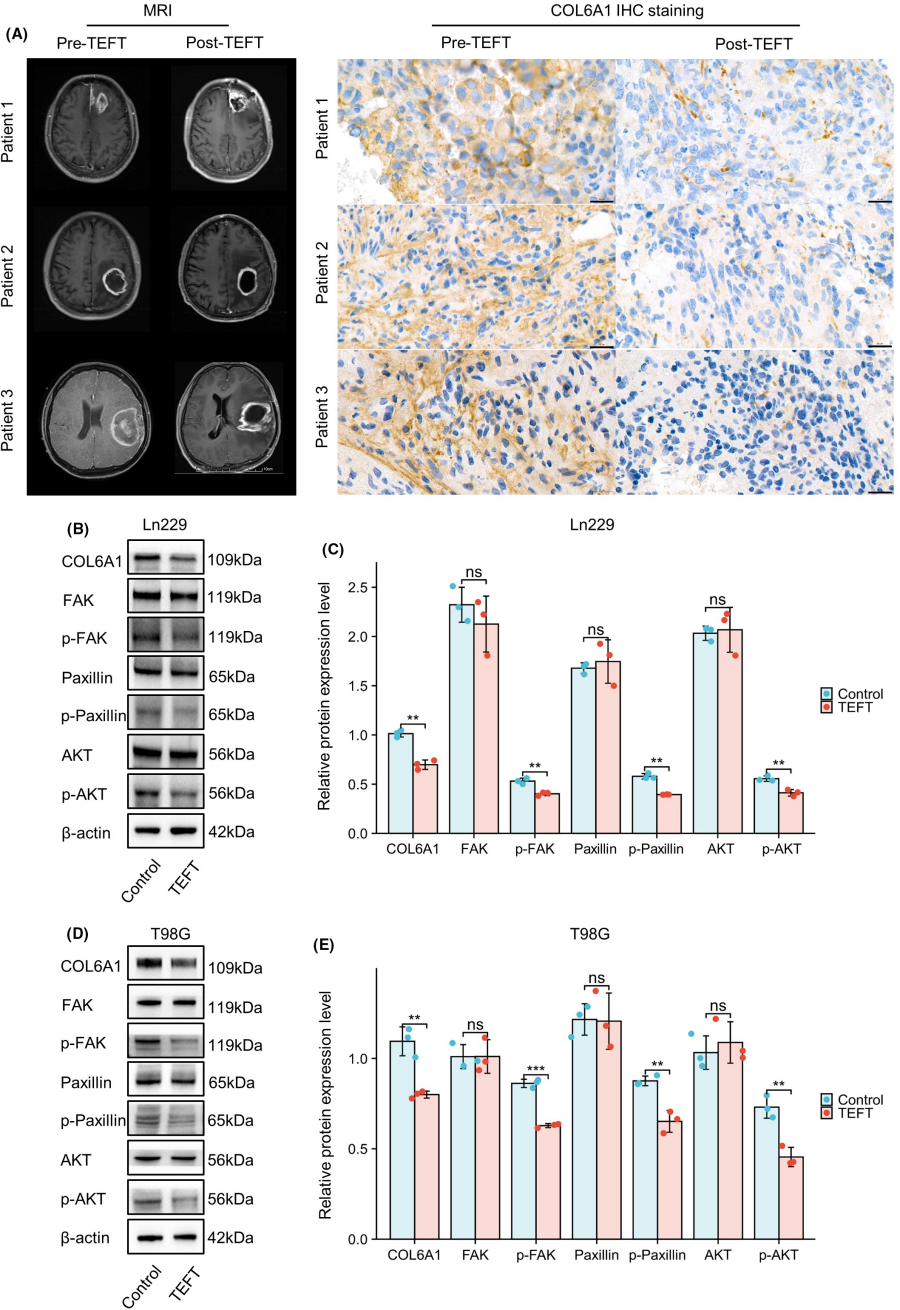
TEFT suppressed COL6A1 expression in GBM patients and cell lines. (A) MRI images of three GBM patients using TEFT. The IHC staining indicated decreased COL6A1 expression after TEFT in all three patients. (B, C) WB exhibited TEFT‐suppressed expression levels of multi‐proteins, including COL6A1, p‐FAK, p‐paxillin, and p‐AKT in the Ln229 cell line. (D, E) WB exhibited TEFT‐suppressed expression levels of multi‐proteins, including COL6A1, p‐FAK, p‐paxillin, and p‐AKT in the T98G cell line. Data are mean ± SD, ns, *p* ≥ 0.05, **p < 0.01, ***p < 0.001, *t*‐test.

Magnetic resonance imaging (MRI) of three GBM patients before and after TEFT showed stable tumor volume after treatment. Immunohistochemical staining for COL6A1 on surgical specimens obtained before and after TEFT therapy demonstrated decreased expression of COL6A1 following treatment in all three patients (Figure 6A). FAK, paxillin, and AKT are critical components of the focal adhesion signaling pathway.^32^, ^33^ Upon activation by integrins, FAK becomes phosphorylated and recruits downstream effectors such as paxillin and AKT, transducing and integrating signals from the extracellular matrix and integrins, ultimately regulating cellular behaviors.^34^, ^35^ WB illustrated that TEFT could significantly downregulate protein level of COL6A1 (**p < 0.01), p‐FAK (**p < 0.01), p‐paxillin (**p < 0.01), and p‐AKT (**p < 0.01) in Ln229 cell line (Figure 6B,C). Additionally, results of T98G cell line were consistent with Ln229 cell line demonstrating downregulation of related proteins including COL6A1 (**p < 0.01), p‐FAK (***p < 0.001), p‐paxillin (**p < 0.01), and p‐AKT (**p < 0.01) (Figure 6D,E). These results pointed out that COL6A1 and focal adhesion pathway might be involved in TEFT‐related antitumor effect.

### 3.7 ITGA5 might be potential integrin molecules binding with COL6A1

**FIGURE 7:**
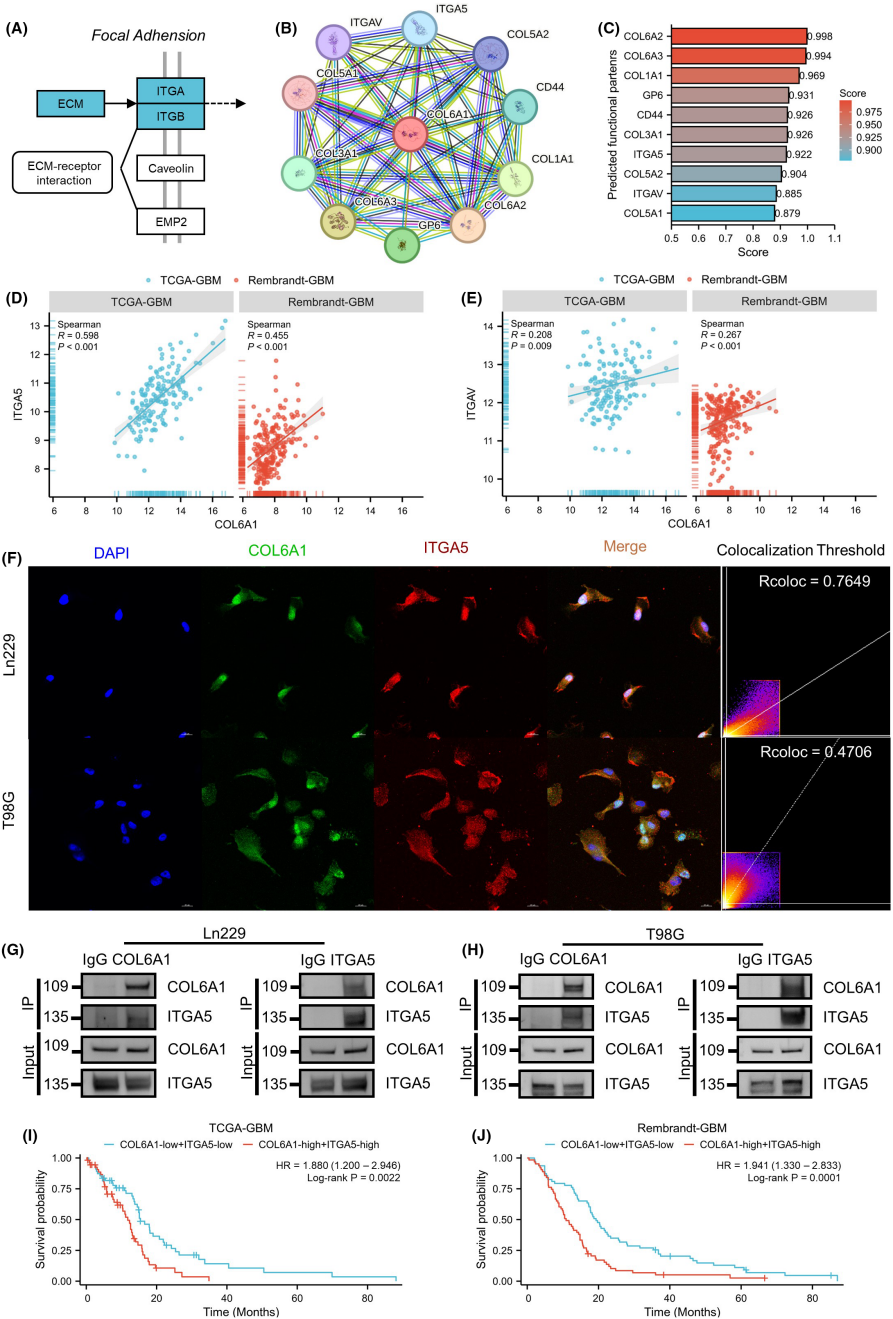
ITGA5 might be potential integrin molecules binding with COL6A1. (A) KEGG map of focal adhesion pathway. (B) PPI network of COL6A1 via STRING database. (C) Predicted functional partners of COL6A1 via STRING database, and different predicting scores are displayed in a gradient color from red to blue. (D) Correlation analysis of COL6A1 and ITGA5 in GBM cohorts (TCGA GBM and Rembrandt GBM), Spearman *r*‐test. (E) Correlation analysis of COL6A1 and ITGAV in GBM cohorts (TCGA GBM and Rembrandt GBM), Spearman r test. (F) Representative IF images and co‐localization analysis of COL6A1 and ITGA5 on Ln229 and T98G cell lines (Ln229: Rcoloc = 0.7649; T98G: Rcoloc = 0.4706). (G, H) Detection of the interaction between COL6A1 and ITGA5 through co‐immunoprecipitation in Ln229 and T98G cell lines. (I) Survival analysis of TCGA GBM patients (COL6A1 high and ITGA5 high vs. COL6A1 low and ITGA5 low), log‐rank test. (J) Survival analysis of Rembrandt GBM patients (COL6A1 high and ITGA5 high vs. COL6A1 low and ITGA5 low), log‐rank test.

Enrichment analysis and WB revealed that focal adhesion pathway was significantly inhibited after TEFT. Given that interplay between ECM and integrins plays important role in focal adhesion (Figure 7A), functional partners of COL6A1 were explored. PPI network detected 10 potential partners, including ITGAV, ITGA5, COL5A2, CD44, COL1A1, COL6A2, GP6, COL6A3, COL3A1, and COL5A1 (Figure 7B). Predicting scores revealed ITGA5 score (0.922) was higher than ITGAV (0.885), indicating binding between COL6A1 and ITGA5 might be more important (Figure 7C). Expression analysis revealed that ITGA5 (TCGA glioma: WHO IV vs. WHO II, ***p < 0.001, WHO IV vs. WHO III, ***p < 0.001; Rembrandt glioma: WHO IV vs. WHO II, ***p < 0.001, WHO IV vs. WHO III, ***p < 0.001) and ITGAV (TCGA glioma: WHO IV vs. WHO II, ***p < 0.001, WHO IV vs. WHO III, *p < 0.05; Rembrandt glioma: WHO IV vs. WHO II, ***p < 0.001, WHO IV vs. WHO III, ***p < 0.001) were highest in GBM (Figure S2A,B). Prognosis analysis revealed that higher expression level of ITGA5 meant shorter OS in Rembrandt GBM cohort (TCGA GBM: hazard ratio = 1.313, 95% confidence interval = 0.918–1.876, *p* = 0.1215; Rembrandt GBM: hazard ratio = 1.450, 95% confidence interval = 1.069–1.965, *p* = 0.0122) (Figure S2C). Additionally, there was no significant correlation between ITGAV expression level and GBM prognosis (Figure S2D). Furthermore, relevance between COL6A1 and ITGA5 (TCGA GBM: Spearman *r* = 0.598, *p* < 0.001; Rembrandt GBM: Spearman *r* = 0.455, *p* < 0.001) was higher than ITGAV (TCGA GBM: Spearman *r* = 0.208, *p* = 0.009; Rembrandt GBM: Spearman *r* = 0.267, *p* < 0.001) (Figure 7D,E). Validation on Ln229 and T98G cell line demonstrated that COL6A1 had significant co‐localization with ITGA5 which suggested that COL6A1 could interact with ITGA5 in GBM (Ln229: Rcoloc = 0.7649; T98G: Rcoloc = 0.4706) (Figure 7F). Furthermore, Co‐IP assays confirmed the protein–protein interaction between COL6A1 and ITGA5 (Figure 7G,H). Combination survival analysis revealed that COL6A1‐high and ITGA5‐high GBM patients had bad prognosis in comparison with COL6A1‐low and ITGA5‐low GBM patients (TCGA GBM: hazard ratio = 1.880, 95% confidence interval = 1.200–2.946, *p* = 0.0022; Rembrandt GBM: hazard ratio = 1.941, 95% confidence interval = 1.330–2.833, *p* = 0.0001) (Figure 7I,J). All these results implied that COL6A1 might interact with ITGA5 and mediate focal adhesion pathway activity.

### 3.8 COL6A1 regulated ITGA5 and its downstream signaling pathways in GBM

**FIGURE 8:**
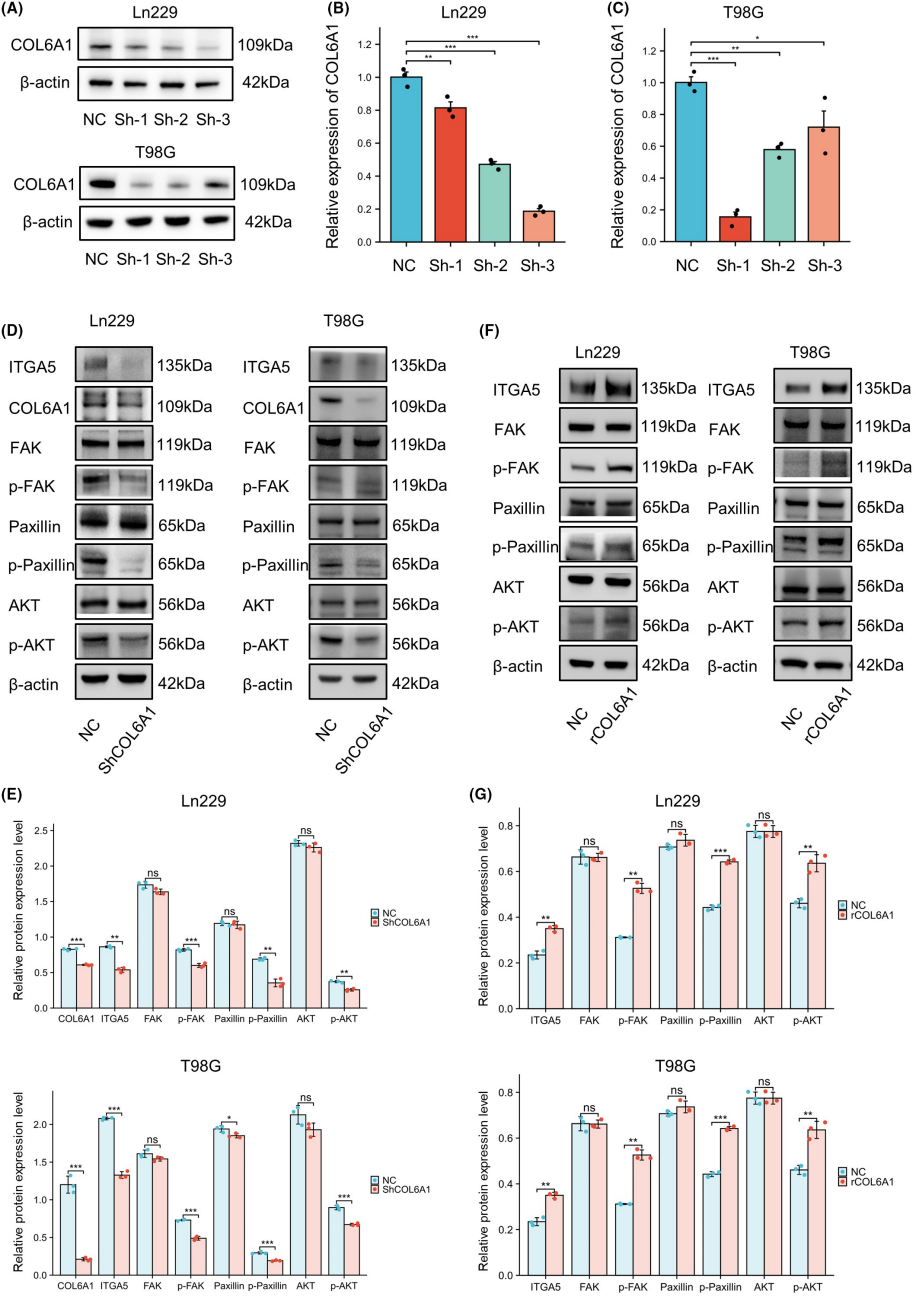
COL6A1‐regulated ITGA5 and its downstream signaling pathways in GBM. (A–C) WB and RT‐qPCR detection of knockout efficiency of COL6A1 gene in Ln229 and T98G cell lines. Data are mean ± SD, no significance (ns), *p* ≥ 0.05, *p < 0.05, **p < 0.01, ***p < 0.001, one‐way ANOVA and Tukey's post‐hoc test. (D,E) WB exhibited that knocking down COL6A1 inhibited the expression levels of various proteins, including ITGA5, p‐FAK, p‐paxillin, and p‐AKT, in Ln299 and T98G cell lines. Data are mean ± SD, ns, *p* ≥ 0.05, *p < 0.05, **p < 0.01, ***p < 0.001, *t* test. (F, G) WB exhibited that the expression levels of ITGA5, p‐FAK, p‐paxillin, and p‐AKT significantly increased after the addition of rCOL6A1 in Ln299 and T98G cell lines. Data are mean ± SD, ns, *p* ≥ 0.05, *p < 0.05, **p < 0.01, ***p < 0.001, *t*‐test.

To further investigate the relationship between COL6A1 and ITGA5, we established stable COL6A1 knockdown cell lines in Ln229 and T98G cells via lentiviral transduction. RT‐qPCR and WB analyses revealed that the Sh‐3 sequence exhibited the highest knockdown efficiency in Ln229 cells, while the Sh‐1 sequence was most effective in T98G cells (Figure 8A–C). Subsequent WB analyses demonstrated that upon COL6A1 knockdown, ITGA5 expression was also suppressed (Ln229: **p < 0.01; T98G: ***p < 0.001), concomitant with decreased phosphorylation levels of its downstream molecules FAK (Ln229: ***p < 0.001; T98G: ***p < 0.001), paxillin (Ln229: **p < 0.01; T98G: ***p < 0.001), and AKT (Ln229: **p < 0.01; T98G: ***p < 0.001) (Figure 8D,E). To verify the function of COL6A1 as an extracellular matrix, we chose to add rCOL6A1 protein to the culture medium. WB results showed that treatment with rCOL6A1 to mimic high‐COL6A1 environment led to a marked upregulation of ITGA5 expression (Ln229: **p < 0.01; T98G: **p < 0.01), accompanied by enhanced phosphorylation of FAK (Ln229: **p < 0.01; T98G: **p < 0.01), paxillin (Ln229: ***p < 0.001; T98G: ***p < 0.001), and AKT (Ln229: **p < 0.01; T98G: **p < 0.01) in both cell lines (Figure 8F,G). Collectively, these findings suggest that COL6A1 may exert its functions in GBM by regulating the ITGA5‐signaling axis and its downstream pathways.

## 4 DISCUSSION

GBM is the most aggressive primary brain tumor with dismal prognosis. In recent years, with the continuous progress of clinical and basic research on GBM, its malignant biological phenotypes and intrinsic mechanisms have been gradually revealed. However, effective therapeutic regimens for GBM are still slowly updated and the prognosis of patients has not been significantly improved.^36^ The emergence of tumor electric field therapy breaks this deadlock. TEFT has shown exciting efficacy in both newly diagnosed and recurrent GBM with few adverse effects,^16^ thus being termed “the fourth modality in cancer treatment.” However, the molecular mechanism of TEFT remains to be further elucidated. In our previous studies, we have validated that cell activity was notably decreased after TEFT, with the proliferation of GBM cells being evidently inhibited.^12^ On this basis, our study further found that TEFT promotes extracellular matrix remodeling of GBM cells, in which COL6A1 was identified as a core gene in this process. TEFT inhibits the expression of COL6A1, interfering with focal adhesion pathways activity, thereby suppressing the viability of GBM cells.

Previous studies have demonstrated that TEFT exerts multifaceted cytotoxic effects on mitotic cells, including classic antimitotic activity as well as inhibition of deoxyribonucleic acid (DNA) replication, induction of autophagy, altered membrane permeability, and enhanced antitumor immunity.^25^, ^37^, ^38^, ^39^, ^40^ The effects of TEFT on the ECM have been rarely explored, and the key genes involved in this process have not been identified. Based on our research group's self‐developed customized TEFT cell intervention system (CL‐301A) and transcriptomic sequencing, the results revealed that numerous ECM components were significantly downregulated following TEFT exposure, indicating ECM remodeling had occurred. Further analyses identified COL6A1 as a potential core gene‐mediating ECM remodeling during this process. To further validate the in vivo effects of TEFT, GBM specimens were collected from patients using our research group's self‐developed wearable TEFT system (ASCLU‐350). Magnetic resonance imaging showed stable tumor volumes after TEFT treatment, consistent with previous studies,^12^, ^15^ further confirming the efficacy of this system. Moreover, immunohistochemical staining indicated that COL6A1 expression was markedly reduced in TEFT‐treated GBM sections compared to untreated controls, implicating COL6A1 as a potential TEFT target.

Collagen VI (COL6) is a unique member of the collagen superfamily with distinct supramolecular assembly and diverse biochemical and cellular protective functions.^41^ COL6 is primarily composed of three polypeptide chains (α1, α2, and α3), with the COL6A1 gene encoding the α1 chain that is often implicated in tumor growth and metastasis.^42^, ^43^, ^44^, ^45^ Several studies have demonstrated that high expression of COL6A1 correlates with poor prognosis in various cancers. In the bladder cancer patient prediction model established by Zhang et al., COL6A1 was a hub gene whose high expression was associated with poorer OS in bladder cancer patients, and the expression of COL6A1 was significantly higher in metastatic bladder cancer tissues compared to non‐metastatic tissues.^42^ Hou et al. found COL6A1 expression was upregulated in cervical cancer tissues and high‐COL6A1 expression significantly correlated with advanced FIGO stage, larger tumor size, lymph node metastasis, and poorer overall and recurrence‐free survival in cervical cancer patients.^43^ Zhang et al. found that COL6A1 overexpression inhibited STAT1 signaling in osteosarcoma cells, promoting migration, invasion, and activation of fibroblasts via packaging into osteosarcoma cell‐derived exosomes to facilitate metastasis.^30^ Owusu et al. showed higher‐COL6A1 expression in pancreatic cancer tissues versus adjacent tissues, and COL6A1 expression was an independent predictor of OS and associated with dismal prognosis.^44^ Glioma‐related studies uncovered that COL6A1 was upregulated in tumor tissues and associated with poor prognosis.^46^, ^47^ Moreover, COL6A1 was found to be differentially expressed across glioma grades, with higher expression levels associated with more advanced tumor grades.^48^ Our results also corroborate previous findings, and we have validated this across multiple datasets at both the mRNA and protein levels. Furthermore, we have also shown that COL6A1 is primarily expressed in tumor cells, with differential expression across tumor cell subtypes. These findings helped us explain the heterogeneity in TEFT efficacy among different patients.

Our study found that TEFT significantly downregulated COL6A1 expression in GBM cells. Transcriptomic sequencing and enrichment analysis revealed that DEGs were mostly enriched in ECM‐related terms. Further protein microarray data analysis showed that the inhibitory effects of COL6A1 on GBM may be associated with ECM‐related functions, particularly some proteins involved in cell adhesion, such as fibronectin, Caveolin 1, IGFBP2, and paxillin, that could promote tumor cell migration, invasion, and survival.^49^, ^50^, ^51^, ^52^, ^53^ The ECM is a crucial core component of all tissues and organs, undergoing constant remodeling and turnover in response to temporal cues and perturbations.^18^ Disruption of normal ECM architecture and homeostasis is often associated with solid tumor initiation and progression.^54^, ^55^ According to Maller et al., tumor‐associated inflammation stimulated collagen crosslinking by stromal fibroblasts, which led to ECM remodeling and stiffening that promoted cancer progression and metastasis.^56^ Goreczny et al.'s study demonstrated that Hic‐5‐mediated remodeling of the tumor stroma ECM by cancer‐associated fibroblasts promoted breast tumor growth, invasion, and metastasis through both biophysical and biochemical mechanisms.^57^ As an important extracellular matrix protein, COL6A1 plays a major role in maintaining ECM homeostasis and architecture.^30^, ^42^, ^58^ Our study identified COL6A1 as a core gene involved in key biological processes that may mediate the antitumor effects of TEFT. Rühl et al. reported that collagen VI stimulates DNA synthesis via increased tyrosine phosphorylation of paxillin and FAK independently of growth factors.^59^ Zhang et al. demonstrated that COL6A1 promoted osteosarcoma cell adhesion to ECM components and increased FAK and Src phosphorylation, implicating COL6A1 in cell adhesion pathways.^30^ Voiles et al. found that COL6A1 activated FAK signaling in lung epithelial cells and macrophages, conferring tumorigenic properties.^60^ Our western blot results illustrated that TEFT treatment not only reduced the expression of COL6A1 but also significantly inhibited the phosphorylation of key focal adhesion proteins such as FAK, paxillin, and AKT, which is consistent with the enrichment analysis, indicating that COL6A1 was closely related to the focal adhesion pathway and might be a novel therapeutic target related to TEFT.

Numerous studies have demonstrated that focal adhesion‐mediated cell–ECM interactions are critical factors in tumor progression and invasion.^61^, ^62^ Within focal adhesions, the interaction between the ECM and integrins is the major pathway.^63^, ^64^ Compared to normal tissues or cells, tumor cells exhibit markedly increased expression of core focal adhesion signaling receptors and specific integrin receptors, and targeting relevant molecules in focal adhesion pathways has proven effective in restoring tumor cell sensitivity to therapies including radiation and chemotherapy.^65^, ^66^ Integrin alpha 5 (ITGA5) is a member of the integrin family of adhesion molecules that play critical roles in cell adhesion and signal transduction and are closely associated with tumor invasion, progression, and chemoresistance.^67^, ^68^ The study by Blandin et al. found that ITGA5 mediated GBM cell diffusion and invasion through cell–matrix and cell–cell interactions.^69^ Li et al. discovered that ITGA5 was involved in remodeling GBM immune infiltration and tumor microenvironment, which were closely related to immunotherapy, and that ITGA5 was a sensitive indicator for a large number of chemotherapeutic drugs.^70^ Furthermore, our previous study showed that ITGA5 expression predicted dual resistance to TMZ and bevacizumab in glioma by promoting vascular mimicry and cell survival.^3^ Our research found that COL6A1 might interact with ITGA5, and regulate the phosphorylation levels of downstream molecules FAK/paxillin/AKT, thereby mediating focal adhesion pathway activity. Moreover, we found that as the expression of COL6A1 changed, ITGA5 also showed the same trend, which is similar to other reported studies. In Xu et al.'s study, after knocking down ITGA5 in the human hepatic stellate cell‐line LX‐2, the expression of COL6A1 was significantly downregulated, and after overexpressing ITGA5, the expression of COL6A1 was also significantly increased.^71^ Shevchenko et al. found that in the human GBM cell‐line U‐87MG, the determinants of focal adhesion ITGA5 and COL6A1 in the cancer stem cells (CSCs) were more than twice as high as in differentiated GBM cells (DGCs).^72^ Based on these findings, we propose that ITGA5 may be a potential therapeutic target for TEFT via interacting with COL6A1. Therefore, we present a promising synthetic therapeutic strategy to use novel small‐molecule inhibitors targeting ITGA5 in combination with TEFT to enhance therapeutic efficacy. Overall, targeting COL6A1 and its signaling networks, either directly or via inhibiting ITGA5, represents a promising therapeutic strategy against TEFT that warrants further research and development.

This study has several limitations that provide avenues for future investigation. First, while we demonstrated that TEFT can remodel the ECM of GBM cells by downregulating the core gene COL6A1, the precise molecular mechanisms involved remain to be fully delineated. Second, the use of immortalized GBM cell lines precluded characterization of in vivo ECM dynamics following TEFT exposure, warranting validation in orthotopic xenograft models to translate findings to the clinical realm. Primary GBM patient‐derived cultures and organoids will also impart critical insights into patient specificity. Detailed delineation of the molecular events bridging TEFT‐induced COL6A1 downregulation to downstream signaling and phenotypes is also needed. Additional limitations pertain to the optimal TEFT frequencies and modalities that were not examined but have been shown to impact efficacy.^12^ Ultimately, comprehensively addressing these limitations will be imperative for advancing our understanding of the anticancer effects of TEFT and their clinical translation.

## 5 CONCLUSION

Our study reveals that TEFT can remodel the ECM of GBM cells and identifies COL6A1 as a core gene. COL6A1 is highly expressed in glioma tissues and associated with clinical prognosis of GBM patients. Therefore, COL6A1 may serve as a novel prognostic biomarker for GBM and a promising new antitumor target for TEFT. Further investigation into the mechanisms of COL6A1 and ECM remodeling in future studies will likely enrich the antitumor mechanisms of TEFT and facilitate the development of TEFT combination therapies.

## AUTHOR CONTRIBUTIONS

JC wrote the manuscript, JC, YL, and JL conducted bioinformatics data analysis and acquired experiment data. All authors discussed the results. HL, QT, ZL, WH, JX, XZ, YF, and LQ contributed to validation and revised the manuscript. JL and LC reviewed, supervised, and acquired funding support. All authors read and approved the final manuscript.

## FUNDING INFORMATION

This work was supported by the National Natural Science Foundation of China (82172680, 82373220, and U20A20380).

## CONFLICT OF INTEREST STATEMENT

The authors declare that they have no conflict of interest. Ling Chen is an Editorial Board Member of CNS Neuroscience and Therapeutics and a co‐author of this article. To minimize bias, they were excluded from all editorial decision‐making related to the acceptance of this article for publication.

## CONSENT

Not applicable.

## Supporting information


**Figure S1.** Multi‐database analysis of COL6A1 expression patterns on single‐cell level. (A–C) Analysis of GSE148842 shows high‐COL6A1 levels in AC‐like malignant and malignant cells. (D–F) Analysis of GSE131928 reveals elevated COL6A1 in AC‐like malignant, MES‐like malignant, malignant, and OPC‐like malignant cells.

**Figure S2.** Expression patterns and prognostic value of ITGA5 and ITGAV in GBM cohorts. (A) Comparison of ITGA5 mRNA levels in glioma with different grades. (B) Comparison of ITGAV mRNA levels in glioma with different grades. Data are mean ± SD, ns, *p* ≥ 0.05, *p < 0.05, ***p < 0.001, Kruskal–Wallis test and Dunn’s post‐hoc test. (C) Survival curves of GBM patients with different ITGA5 expression levels (ITGA5 high vs. ITGA5 low). (D) Survival curves of GBM patients with different ITGAV expression levels (ITGAV high vs. ITGAV low), log‐rank test.

**Table S1.** The short hairpin RNA‐targeting COL6A1 for lentiviral construction in this study.

**Table S2.** Primers used in this study.

**Table S3.** Antibodies used in this study.

**Data S1.** Supporting Information.

## Data Availability

The datasets used and analyzed during the current study are available from the corresponding author upon reasonable request.
