# Supplementary figures and images for: Identification and validation of COL6A1 as a novel target for tumor electric field therapy in glioblastoma
Source: CNS Neurosci Ther. 2024 Jun 17;30(6):e14802. doi: 10.1111/cns.14802 (PMC11183175; doi:10.1111/cns.14802)

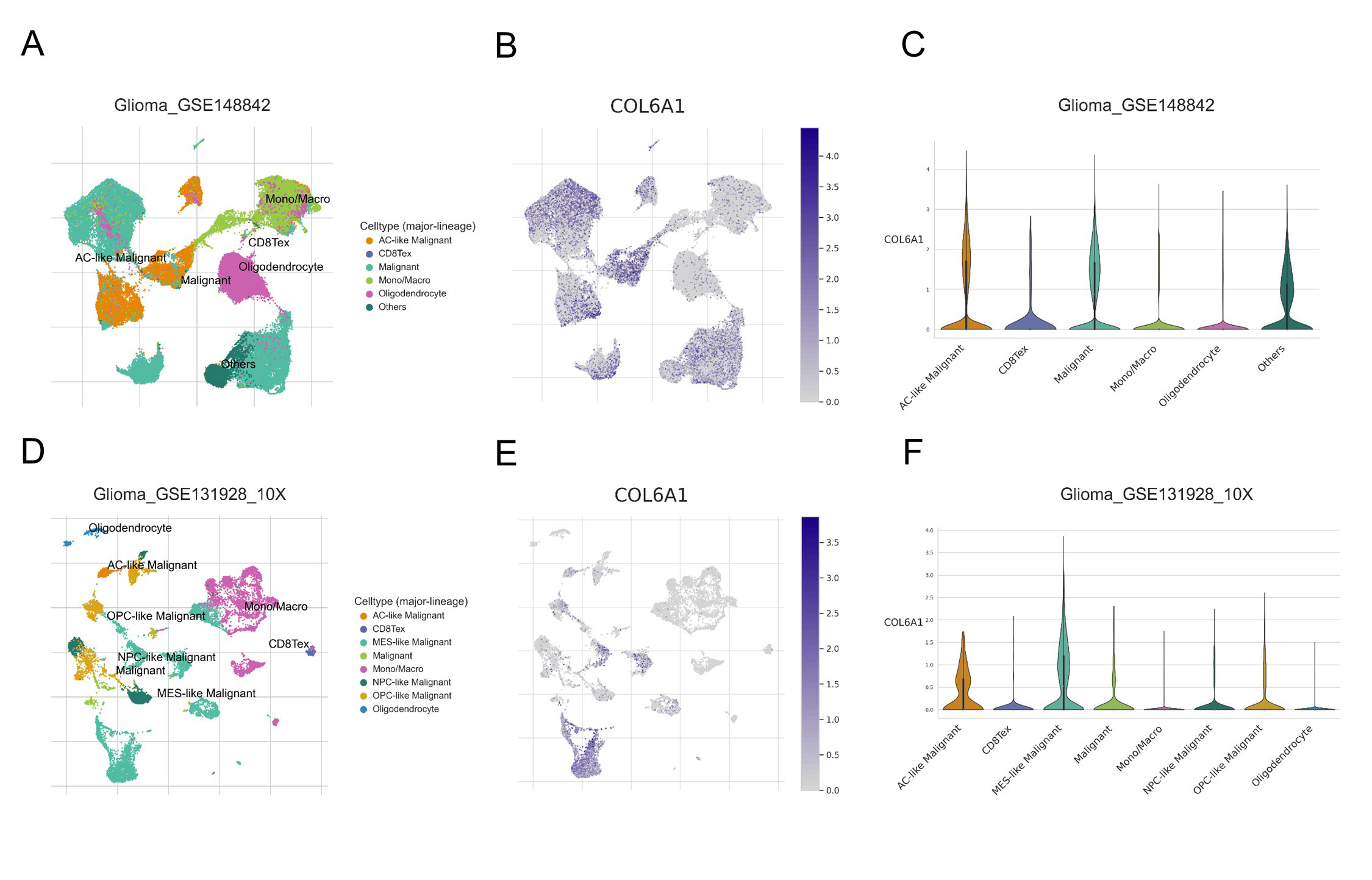

Supplement: Supplementary file 1 — Figure S1. Multi‐database analysis of COL6A1 expression patterns on single‐cell level. (A–C) Analysis of GSE148842 shows high‐COL6A1 levels in AC‐like malignant and malignant cells. (D–F) Analysis of GSE131928 reveals elevated COL6A1 in AC‐like malignant, MES‐like malignant, malignant, and OPC‐like malignant cells. [file CNS-30-e14802-s004.jpg]

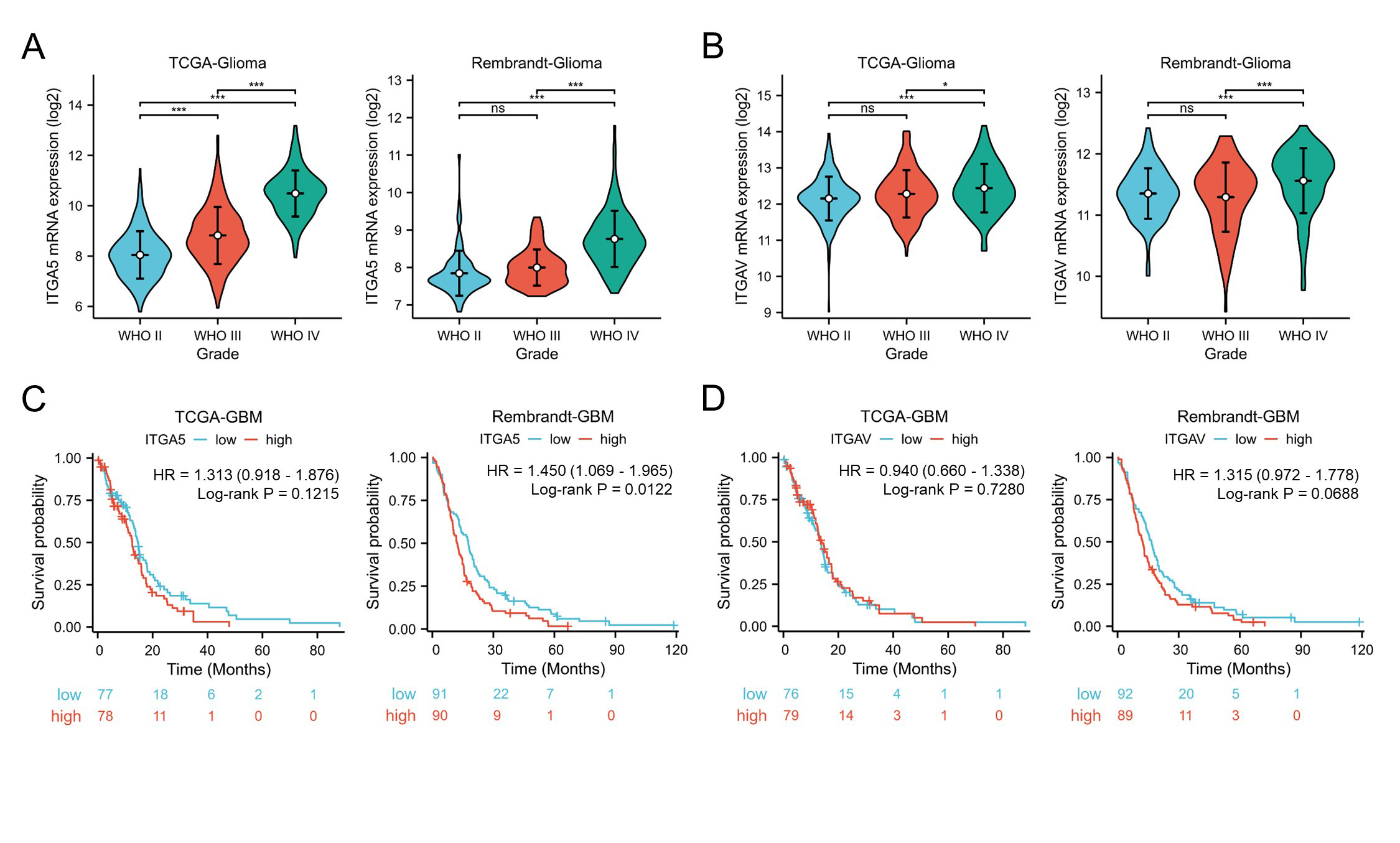

Supplement: Supplementary file 2 — Figure S2. Expression patterns and prognostic value of ITGA5 and ITGAV in GBM cohorts. (A) Comparison of ITGA5 mRNA levels in glioma with different grades. (B) Comparison of ITGAV mRNA levels in glioma with different grades. Data are mean ± SD, ns, p ≥ 0.05, *p < 0.05, ***p < 0.001, Kruskal–Wallis test and Dunn’s post‐hoc test. (C) Survival curves of GBM patients with different ITGA5 expression levels (ITGA5 high vs. ITGA5 low). (D) Survival curves of GBM patients with different ITGAV expression levels (ITGAV high vs. ITGAV low), log‐rank test. [file CNS-30-e14802-s002.jpg]
