# Supplementary material for: Identification and validation of COL6A1 as a novel target for tumor electric field therapy in glioblastoma
Source: CNS Neurosci Ther. 2024 Jun 17;30(6):e14802. doi: 10.1111/cns.14802 (PMC11183175; doi:10.1111/cns.14802)
Supplement: Supplementary file 3 — Table S1. The short hairpin RNA‐targeting COL6A1 for lentiviral construction in this study. [file CNS-30-e14802-s005.docx]

**Table S1. The Short hairpin RNA targeting COL6A1 for lentiviral construction in this study**

| ShCOL6A1 -1 | CCGGGGAGAATAAGTACCTGATTGTCTCGAGACAATCAGGTACTTATTCTCCTTTTTT |
| --- | --- |
| ShCOL6A1 -2 | CCGGGCCTGCAGAACTTCGAGATTGCTCGAGCAATCTCGAAGTTCTGCAGGCTTTTTT |
| ShCOL6A1 -3 | CCGGGTGGGCATCAAAGACGTGTTTCTCGAGAAACACGTCTTTGATGCCCACTTTTTT |
