# Supplementary material for: Identification and validation of COL6A1 as a novel target for tumor electric field therapy in glioblastoma
Source: CNS Neurosci Ther. 2024 Jun 17;30(6):e14802. doi: 10.1111/cns.14802 (PMC11183175; doi:10.1111/cns.14802)
Supplement: Supplementary file 4 — Table S2. Primers used in this study. [file CNS-30-e14802-s006.docx]

**Table S2. Primers used in this study**

| **Gene** | **Forward sequence (5' – 3')** | **Reverse sequence (5' – 3')** |
| --- | --- | --- |
| RT-qPCR primers | | |
| COL6A1 | CACTCAAAAGCAGCGTGGAC | GTCGGTCACCACAATCAGGT |
| Actin | CCTCACCCTGAAGTACCC | AGCCTGGATAGCAACGTACATG |
