# Supplementary material for: Identification and validation of COL6A1 as a novel target for tumor electric field therapy in glioblastoma
Source: CNS Neurosci Ther. 2024 Jun 17;30(6):e14802. doi: 10.1111/cns.14802 (PMC11183175; doi:10.1111/cns.14802)
Supplement: Supplementary file 5 — Table S3. Antibodies used in this study. [file CNS-30-e14802-s001.docx]

**Table S3. Antibodies used in this study**

| Antibody | Application | Host | Supplier |
| --- | --- | --- | --- |
| COL6A1 | WB and IP | Rabbit | Cat:# NB120-6588, NOVUS |
| FAK | WB | Rabbit | Cat:# AF6397, Affinity Biosciences |
| p-FAK | WB | Rabbit | Cat:# AF3398, Affinity Biosciences |
| AKT | WB | Rabbit | Cat:# AF6261, Affinity Biosciences |
| p-AKT | WB | Rabbit | Cat:# AF0016, Affinity Biosciences |
| Paxillin | WB | Rabbit | Cat:# AF6332, Affinity Biosciences |
| p-Paxillin | WB | Rabbit | Cat:# AF3933, Affinity Biosciences |
| ITGA5 | WB, IF and IP | Rabbit | Cat:# AB1928, Merk |
| β-actin | WB | Rabbit | Cat:# 4970, Cell Signaling Technology |
| COL6A1 | IF | Mouse | Cat:# SC377143, Santa |
