# Supplementary material for: Identification and validation of COL6A1 as a novel target for tumor electric field therapy in glioblastoma
Source: CNS Neurosci Ther. 2024 Jun 17;30(6):e14802. doi: 10.1111/cns.14802 (PMC11183175; doi:10.1111/cns.14802)
Supplement: Supplementary file 6 — Data S1. Supporting Information. [file CNS-30-e14802-s003.pdf]

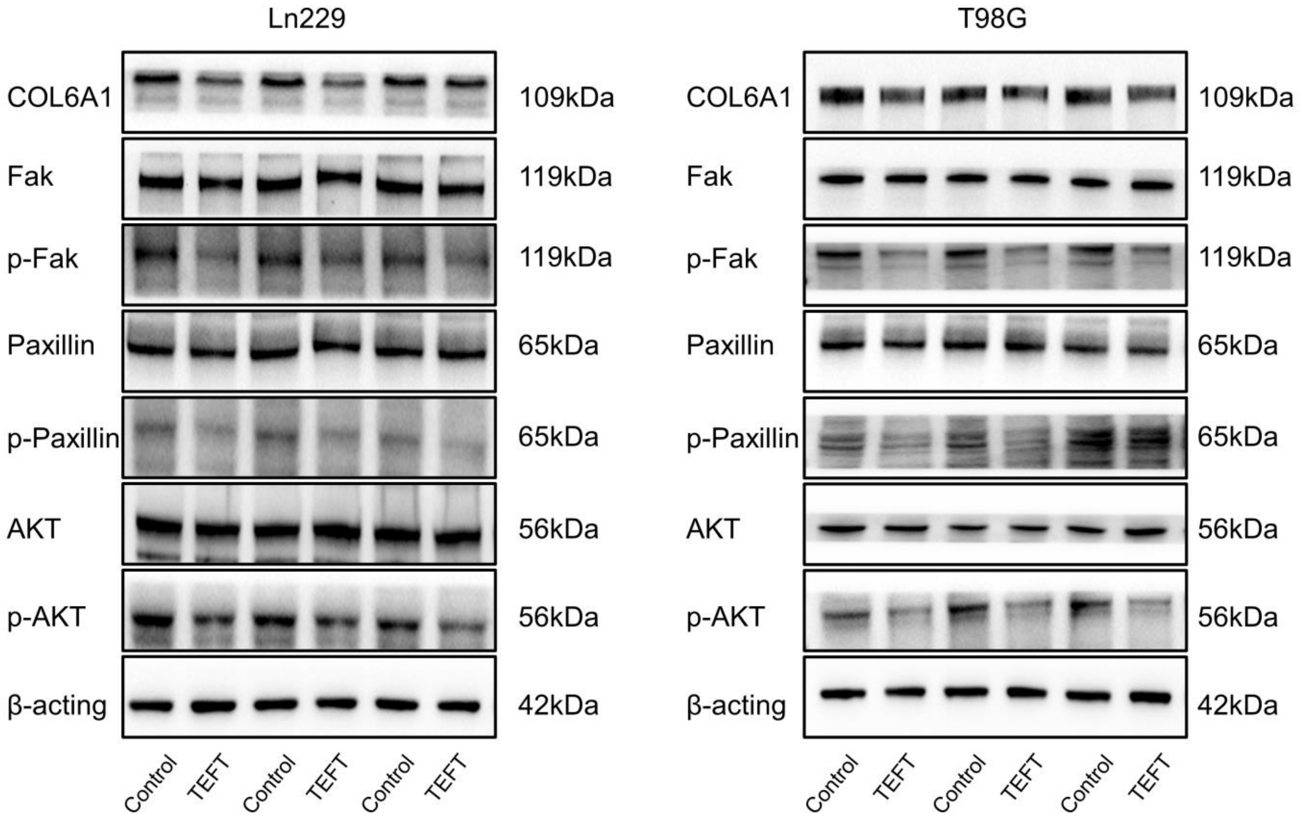

Full unedited blot for Figure 6B and D

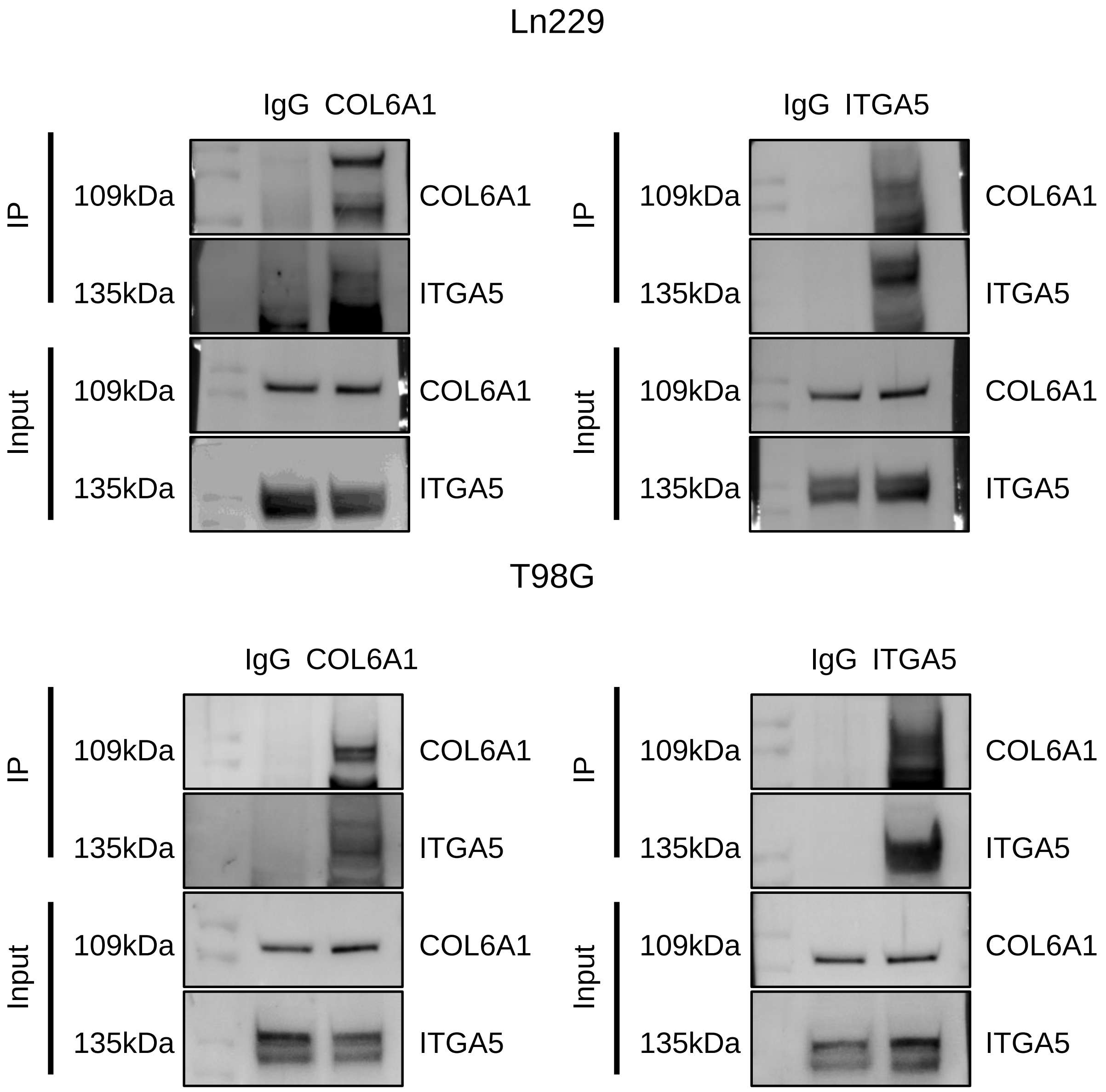

Full unedited blot for Figure 7G and H

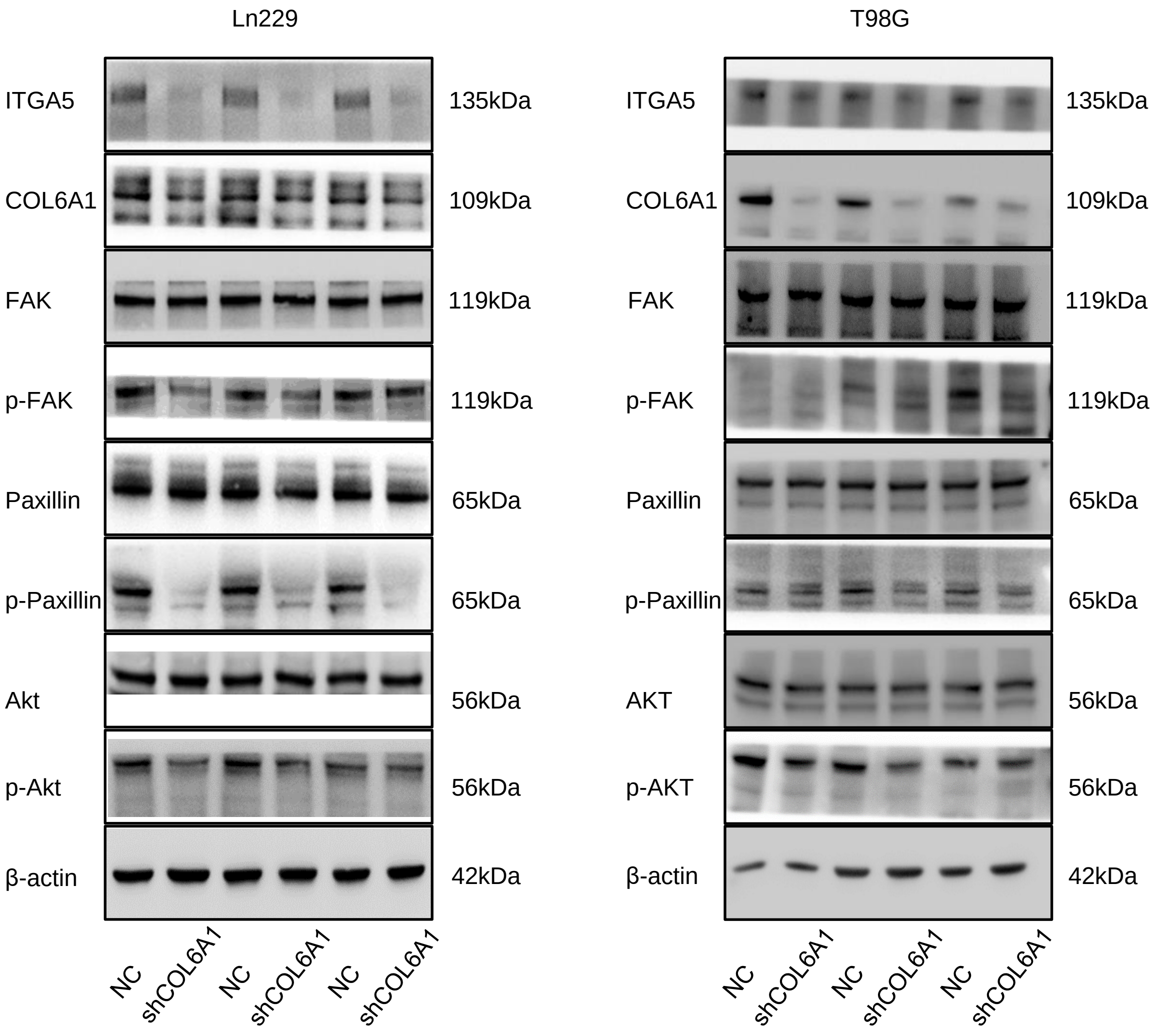

Full unedited blot for Figure 8D

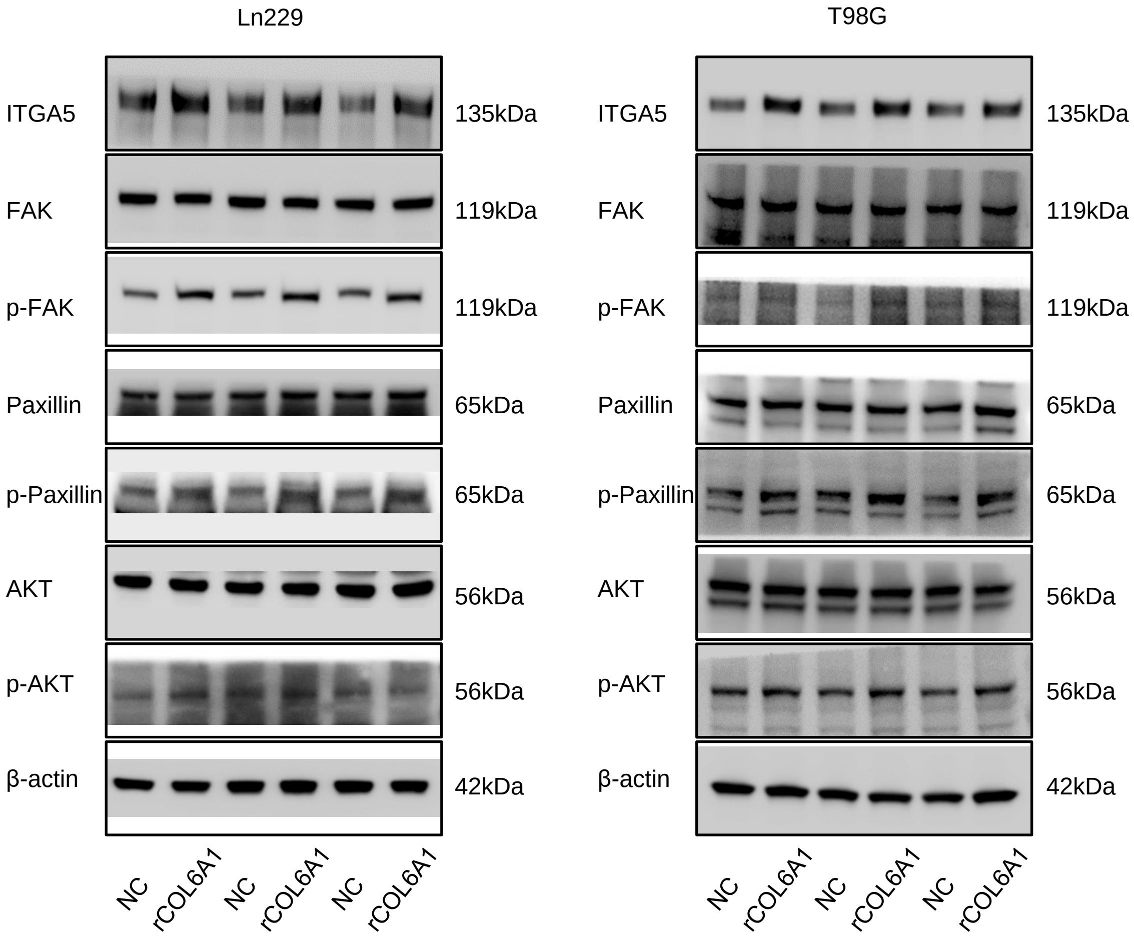

Full unedited blot for Figure 8F

Table S1. The Short hairpin RNA targeting COL6A1 for lentiviral construction in this study

**ShCOL6A1 -1**	CCGGGGAGAATAAGTACCTGATTGTCTCGAGACAATCAGGTACTTATTCTCCTTTTTT
**ShCOL6A1 -2**	CCGGGCCTGCAGAACTTCGAGATTGCTCGAGCAATCTCGAAGTTCTGCAGGCTTTTTT
**ShCOL6A1 -3**	CCGGGTGGGCATCAAAGACGTGTTTCTCGAGAAACACGTCTTTGATGCCCACTTTTTT

Table S2. Primers used in this study

**Gene**	**Forward sequence (5' – 3')**	**Reverse sequence (5' – 3')**
**RT-qPCR primers**		
**COL6A1**	CACTCAAAAGCAGCGTGGAC	GTCGGTCACCACAATCAGGT
**Actin**	CCTCACCCTGAAGTACCC	AGCCTGGATAGCAACGTACATG

Table S3. Antibodies used in this study

Antibody	Application	Host	Supplier
**COL6A1**	WB and IP	Rabbit	Cat:# NB120-6588, NOVUS
**FAK**	WB	Rabbit	Cat:# AF6397, Affinity Biosciences
**p-FAK**	WB	Rabbit	Cat:# AF3398, Affinity Biosciences
**AKT**	WB	Rabbit	Cat:# AF6261, Affinity Biosciences
**p-AKT**	WB	Rabbit	Cat:# AF0016, Affinity Biosciences
**Paxillin**	WB	Rabbit	Cat:# AF6332, Affinity Biosciences
**p-Paxillin**	WB	Rabbit	Cat:# AF3933, Affinity Biosciences
**ITGA5**	WB, IF and IP	Rabbit	Cat:# AB1928, Merk
**β-actin**	WB	Rabbit	Cat:# 4970, Cell Signaling Technology
**COL6A1**	IF	Mouse	Cat:# SC377143, Santa
